# The Role of Event Number and Duration in Time-Compressed Memory Replay

**DOI:** 10.1162/OPMI.a.276

**Published:** 2025-12-18

**Authors:** Nathan Leroy, Arnaud D’Argembeau

**Affiliations:** Psychology and Neuroscience of Cognition Research Unit, University of Liège, Liège, Belgium

**Keywords:** temporal compression, event segmentation, duration, episodic memory

## Abstract

Remembering the unfolding of past experiences usually takes less time than their actual duration. In this study, we examined the extent to which this temporal compression in memory depends on the number and duration of events that need to be maintained in a sequence. Participants were asked to watch and then mentally replay short videos depicting one, two, or three continuous events (i.e., people performing continuous actions in an uninterrupted way), each lasting 3, 6, 9, or 12 s. Across two experiments, we computed indices of remembering duration and temporal compression for each event. Results showed that event remembering duration was close to the actual event duration for short events, but smaller for longer ones (i.e., temporal compression was not systematic but occurred selectively depending on event duration). Furthermore, events were mentally replayed more quickly when they were part of a sequence of several events than when they were presented alone, and this decrease in the duration of event recall with the number of events was more pronounced for longer events. Exploratory analyses revealed that individual differences in memory compression were predicted by visual imagery capacity. These results suggest that working memory capacity in representing naturalistic events is limited by both the number and duration of events to be retained, which may in part explain why the unfolding of events is temporally compressed in episodic memory.

## INTRODUCTION

In the course of daily life, we experience a continuous flow of information from our senses and mental activity. Episodic memory registers this information stream, enabling us to mentally relive our past experiences (Tulving, 2002). However, our memories are not like continuous video recordings (Conway, 2009), but instead represent the unfolding of events in a time-compressed form (D’Argembeau et al., 2022). Indeed, remembering usually takes far less time than the actual duration of the past experience (Bonasia et al., 2016; Chen et al., 2017; Faber & Gennari, 2015; Jeunehomme & D’Argembeau, 2019). For example, it may take you a few minutes to remember the party you threw for your friend’s birthday last weekend, when in reality it lasted several hours. Although this temporal compression is a central aspect of everyday memories, the underlying mechanisms remain unclear. To shed light on this issue, we sought to determine the extent to which temporal compression rates in memory representations depend on the microstructure of event sequences, namely the number and duration of events to be stored.

Recent research into the temporal structure of memories of real-world events has shown that the unfolding of events is remembered as a sequence of units of experience, each unit representing a moment or segment of the past episode (for a review, see D’Argembeau et al., 2022). Importantly, however, this sequence of experience units is not continuous, but contains temporal discontinuities: some segments of past experience are omitted when the course of events is mentally replayed (Jeunehomme & D’Argembeau, 2023; Jeunehomme et al., 2018; Michelmann et al., 2023). The cognitive mechanisms leading to this structure of episodic memories are not fully understood, but research suggests that temporal discontinuities in memory representations may result from the way we integrate the continuous flow of experience in long-term memory by breaking it down into discrete units (i.e., events and sub-events; Bird, 2020; Jeunehomme & D’Argembeau, 2020; Loschky et al., 2020; Zacks, 2020). According to event segmentation theory (Zacks et al., 2007), to make sense of ongoing experience, we continually construct a mental model of the current situation (i.e., an event model), which is maintained in working memory (WM; Richmond & Zacks, 2017). When significant perceptual or conceptual changes occur, an event boundary is perceived—the subjective experience that an event has ended, and another begins—and the event model maintained in WM is updated and transferred into long-term memory (Baldassano et al., 2017; Kurby & Zacks, 2008; Loschky et al., 2020; Wang et al., 2024).

The mechanism of encoding of event models proposed by event segmentation theory suggests that the experience units that are formed in long-term memory may depend on WM capacity (Güler et al., 2024; Leroy et al., 2025 Sargent et al., 2013). When the information accumulated in an event model exceeds WM capacity, the mental representation of the event’s unfolding may start to be truncated (i.e., part of the sensory stream would no longer be represented), leading to incomplete memory encoding of within-event information in long-term memory. A recent study that assessed temporal compression in memory for continuous events varying in duration supports this view (Leroy et al., 2024). Participants were presented with a series of videos, each showing a continuous event (with no event boundary) that lasted from 3 to 15 s. Immediately after the presentation of each video, participants had to mentally replay the event’s unfolding while the time needed to do so was recorded. The results showed that the duration of mental replay closely followed the actual duration of the event for short events, but began to be time-compressed when events lasted longer than 9 s. These results suggest that WM is temporally limited in its capacity to represent continuous events, which could in part explain why the unfolding of events is temporally compressed in episodic memory representations.

This research on the effect of event duration on memory replay for continuous events sheds a first light on the mechanisms by which some within-event information can be lost when event models are transferred to long-term memory. However, this is probably only one piece of the puzzle. Beyond the duration of events, other aspects of the microstructure of the continuous flow of experience could shape the temporal resolution with which events are represented in memory. The perception of an event boundary does not always result in the transfer of the current event model to long-term memory. Indeed, event segmentation is known to operate simultaneously at multiple time scales and levels of specificity, leading to a hierarchy of event representations: groups of fine-grained events cluster into larger event units (Hard et al., 2011; Zacks, 2020). For example, the event “make coffee” can be broken down into sub-events such as “choose a capsule”, “put the capsule in the machine”, “place the cup”, “start the machine”, and so on. The transfer of WM content to episodic memory is triggered by the perception of high-level or coarse event boundaries (e.g., making coffee vs. checking emails), whereas lower-level or fine event boundaries (e.g., choosing a capsule vs. putting the capsule in the machine) would indicate the accumulation of information in the ongoing event model constructed in WM (Baldassano et al., 2017; Bird, 2020; Huff et al., 2014; Kurby & Zacks, 2008). Therefore, in many situations, it is not just a single event but a sequence of several events that needs to be maintained in WM, until the content of WM is transferred to long-term memory. We propose that the processing of such event sequence involves the formation of chunks in WM.

The role of chunking in optimizing the maintenance of information in WM is well known (Norris & Kalm, 2021). Chunking can be framed as a form of data compression, allowing more elements to be maintained in less space by removing redundant information: regularities in perceptual input are used to form more compact representations (Brady et al., 2009; Mathy et al., 2024; Norris & Kalm, 2021). In support of this view, research has shown that the number of items that can be maintained in WM increases when they contain statistical regularities, as highly correlated items can be summarized in a single chunk (Brady et al., 2009; Chekaf et al., 2016; Kowialiewski et al., 2022; Lemaire et al., 2012; Norris et al., 2020). However, although the creation of chunks in WM enables a higher number of items to be remembered, the amount of information that can be maintained (i.e., WM capacity) remains unchanged, so this increase in the number of retained items comes at the expense of memory precision. Indeed, highly redundant items tend to be remembered with less precision (Al Roumi et al., 2021; Lazartigues et al., 2021; Mathy et al., 2024; Nassar et al., 2018; Ramzaoui & Mathy, 2021).

A similar chunking mechanism might operate when processing naturalistic events. In fact, event segmentation can be thought as a temporal chunking of the perceptual stream, leading to the formation of compressed “high-predictability event units” (Baldwin & Kosie, 2021; McGatlin et al., 2019) that are created by the integration vs. segregation of information in the sensory stream (Clewett & Davachi, 2017; Clewett et al., 2019). When experiencing a sequence of events, fine event boundaries may induce the chunking of information that needs to be maintained in WM: each boundary may trigger the creation of a WM representation that synthetically represents the unfolding of the just-experienced event, freeing up WM resources for the maintenance of subsequent events. The reduced precision of representations following chunking may in part explain the time-compressed replay of naturalistic events that are maintained in WM. Consequently, although the total remembering duration of a sequence should increase with the number of events it contains, each event, taken individually, should be remembered more quickly (less completely) when it is encoded as part of a sequence than if it is presented in isolation. In addition, as longer events are likely to involve more redundancies (i.e., their unfolding is more compressible), the decrease of event temporal resolution with the number of events should increase with event duration.

In summary, the formation of experience units in episodic memory may be shaped by the microstructure of the event model that is maintained in WM to make sense of ongoing experience. In particular, the temporal resolution with which events are represented may depend on the number and duration of events that need to be maintained in a sequence. We here tested this hypothesis in two experiments.

## EXPERIMENT 1

In Experiment 1, we asked participants to watch and mentally replay videos depicting sequences of lifelike events (i.e., continuous actions performed without interruption), and we manipulated the number and duration of events included in the sequences. For each trial, participants were asked to mentally replay the sequence of events, in as much detail as possible, and we measured the time it took them to remember it. Based on this measure, we computed two indices estimating the temporal resolution of memory for the individual events that composed the sequence: the event remembering duration (i.e., the total memory replay duration divided by the number of events in the video) and the event temporal ratio (i.e., the ratio between event remembering duration and the actual event duration). First, we aimed to replicate previous results (Leroy et al., 2024) showing that, when events are presented in isolation, event remembering duration increases non-linearly with event duration, with temporal compression emerging for longer events (i.e., the temporal ratio should be close to 1 for short events, then lower for long events). Second, we predicted that when several events are presented, the perceptual stream is temporally chunked to allow representing the entire sequence in WM, such that memory for the individual events composing the sequence is shortened (i.e., event remembering duration and temporal ratio decrease). Insofar as the maintenance of several events in WM is made possible by a drop in within-event information, the effect of the number of events on the two temporal resolution indices should be greater for sequences composed of longer events.

In addition to addressing these main research questions, we also aimed to explore the relationship between individual differences in the vividness of visual mental imagery and the temporal compression of events in memory. Given the close links between visual imagery and the maintenance of items in visual WM (Albers et al., 2013; Baddeley & Andrade, 2000; Ceja & Franconeri, 2023; Keogh & Pearson, 2011), we expected that people who have more vivid visual imagery would display lower memory compression rates when mentally replaying events. Furthermore, to the extent that the effects of event number and duration on memory compression result from WM capacity limit, we hypothesized that these effects would be reduced for individuals with higher vividness of visual imagery. These hypotheses, the experimental design, and analysis plan were preregistered in OSF at https://osf.io/a6ydj.

### Methods

#### Participants.

Participants were 72 young adults (32 women and 40 men) aged between 18 and 35 years (*M* = 23.6, *SD* = 7) who were recruited through announcements on social media and word-of-mouth. To be included, participants had not to be currently taking any medication that could affect their ability to concentrate or have a history of psychiatric, psychological, or neurological disorder. Our main statistical analyses were conducted using linear mixed-effects models (Brauer & Curtin, 2018), and the targeted sample size was determined using a power analysis based on Monte-Carlo simulations (Brysbaert & Stevens, 2018; DeBruine & Barr, 2021). We conducted a pilot study with 10 participants (who were not included in our final sample) and used these pilot data to fit each statistical model we planned to run on our final dataset. Then, we computed a power curve (showing the statistical power that can be achieved with a range of sample sizes) for each of these models. The alpha level was set to 0.05. Taken together, these analyses indicated that a sample size of 50 participants would provide a statistical power of at least 90% to detect the effects of interest (pilot data, scripts, and power analyses are available at https://osf.io/6aecj). To have an equal number of participants assigned to each of our 24 sets of stimuli (see below), we targeted a sample size of 72 participants. All participants provided written informed consent, and the study was approved by the local ethics committee (ref. 2122-009).

#### Materials and Procedure.

**Figure 1.**
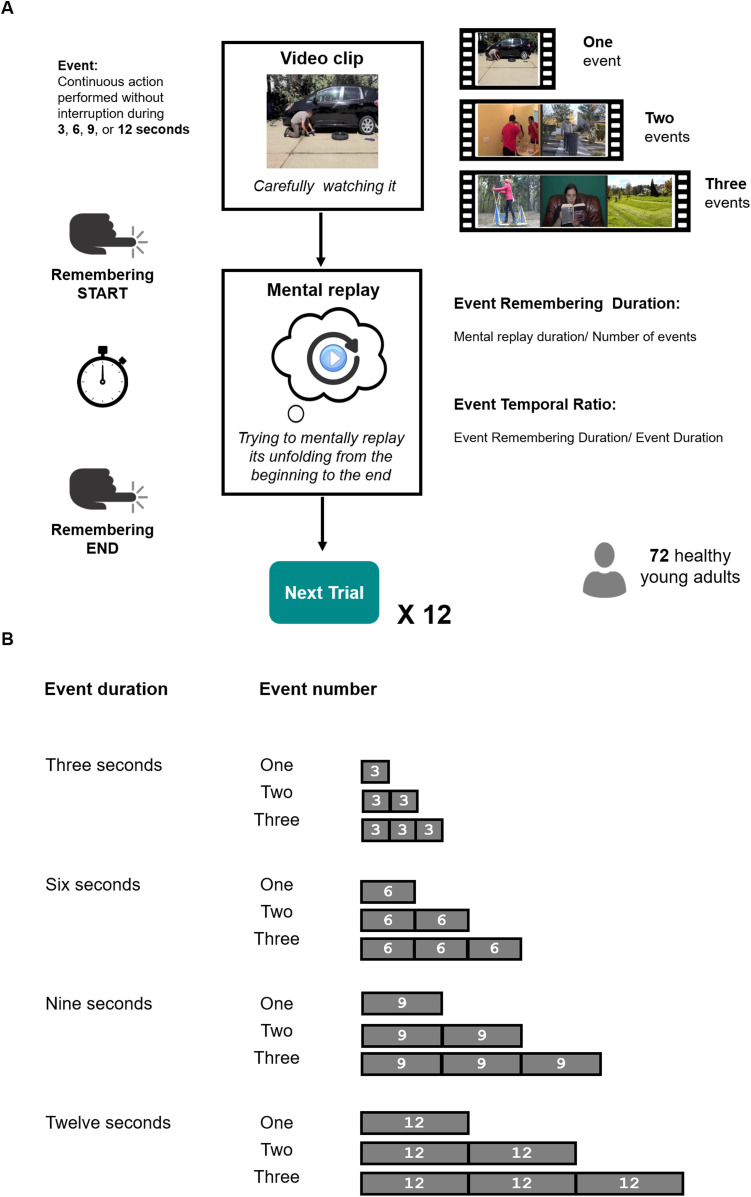
Summary of the experimental paradigm. *Note*. (**A**) *Experimental design and trial structure*. Each trial started with a video clip composed of either one, two or three events. Each event depicted a person engaged in a continuous action (performed without interruption) during 3, 6, 9, or 12 s. As soon as the video ended, participants had to mentally replay its unfolding in as much detail as possible, as if they saw again the depicted event(s). They were asked to press the spacebar when they started and when they finished their mental replay, allowing us to estimate the time they took to mentally replay the video clips. Based on this measure, we computed two indices: the event remembering duration (i.e., the mental replay duration divided by the number of events in the clip) and the event temporal ratio (i.e., the ratio between event remembering duration and event duration). (**B**) *Features of the stimuli*. Each participant had to perform 12 trials of the mental replay task. Each trial corresponded to one of the 12 possible combinations of our variables of interest: event duration and event number (e.g., 3-s event presented alone, 3-s event presented in sequence of two events, 3-s event presented in a sequence of three events, 6-s event presented alone, and so on).

Participants had to mentally replay a series of videos depicting people performing daily life activities (see Figure 1). To examine how temporal compression in memory replay is impacted by the number and duration events, each participant was exposed to 12 videos that included different numbers of events (one, two or three) of different durations (3, 6, 9, or 12 s).

The stimuli were constructed based on 24 movie clips showing one (or several) person(s) performing a continuous action (e.g., turning a car jack) for at least 12 s, with no event boundary (i.e., each video showed a single continuous action). Some of the movie clips were selected from previous studies on event segmentation (Eisenberg & Zacks, 2016; Kurby & Zacks, 2011; Sargent et al., 2013; Smith et al., 2020, 2021; Wahlheim et al., 2022) and others were downloaded from a website hosting free-to-use audiovisual content (https://pixabay.com/). Each movie clip was edited to construct versions of the same event that lasted 3, 6, 9, and 12 s^1^. The 3-s version corresponded to the first 3 s of the event, the 6-s version corresponded to the first 6 s of the event, and so on. Then, for each event duration, we created video stimuli containing one, two or three events^2^. In total, we constituted 24 sets of stimuli, each containing 12 videos (4 event durations × 3 numbers of events), such that the assignment of an action to a given event number, event duration or position in the sequence (e.g., first in a stimulus containing two events, third in a stimulus containing three events, etc.) was counterbalanced across participants. Each participant was randomly assigned to one of these 24 sets (with replacement). Within each set, the order of trials was fully randomized. All the video stimuli as well as the spreadsheet that ruled their order of presentation are available in OSF at https://osf.io/6aecj.

Each trial started with a fixation cross of 3 s, followed by a video. Participants were instructed to watch the video carefully and then to mentally replay it, as accurately and precisely as possible (as if they were watching the video again in their minds). The time taken by participants to mentally replay the unfolding of the video was measured by asking them to press the spacebar to indicate the beginning and end of remembering (Jeunehomme & D’Argembeau, 2019)^3^. Participants performed the task on a laptop via the Gorilla platform (https://gorilla.sc/) and were supervised by the experimenter during the entire testing. The detailed instructions are available in OSF at https://osf.io/6aecj.

To familiarize participants with the procedure, they performed three practice trials (one for each number of events) before starting the main task. Events displayed in these practice trials were not used for the main task. During this training session, the experimenter ensured that the concept of mental replay was well understood by the participant. For each trial, the participant was asked to explain what was happening in their mind while they mentally replayed the video, and the experimenter checked that they attempted to mentally relive the event’s unfolding in as much detail as possible and that they made the key presses to indicate the beginning and end of their mental replay at the correct moments. If necessary, the experimenter re-explained the instructions to clarify what was expected of them. The training was repeated until the participant understood and applied the instructions correctly. This training session aimed to ensure that the time elapsed between the participant’s two key presses actually reflected the time taken to mentally re-visualize the unfolding of the video (i.e., the duration of the mental replay).

After the memory task, participants’ vividness of visual imagery was assessed using the Vividness of Visual Imagery Questionnaire (VVIQ), which comprises 16 items referring to different situations that the participant is asked to mentally visualize (Marks, 1973). For each item, participants had to rate their mental images on a 5-point scale according to their vividness (1 = no image, 5 = as vivid as perception)^4^. We used a French version of the VVIQ (Santarpia et al., 2008). Participants could either keep their eyes open or close them (no instruction was given in this respect, as in the mental replay task; Pearson et al., 2011). We computed a total VVIQ score by summing the 16 VVIQ ratings. The questionnaire showed a good reliability in our sample (Cronbach’s alpha = 0.79, bootstrapped 95% CI [0.69, 0.85]; see Supplementary Materials for more detail).

#### Data Cleaning.

Data cleaning was performed following our preregistered plan (https://osf.io/a6ydj). First, participants who did not complete the entire memory task were excluded from the analyses, and trials for which there was a technical issue with the video presentation (e.g., an abnormal duration of presentation) were excluded. In addition, we removed trials with a time of mental replay shorter than 1 s (i.e., to remove trials during which the participant inadvertently pressed the button twice or did not properly follow the instructions) or longer than twice the real duration of the remembered video (i.e., to remove trials during which the participant was interrupted or did not mentally replay the video in one shot). If more than half of the trials of one type (one duration) had to be excluded for a given participant, the entire participant was removed from the analyses. Each removed participant was replaced by another participant. In total, 6 participants were removed. The analyses reported here are based on 849 observations from 72 participants.

#### Statistical Analyses.

All statistical analyses were performed using R (version 4.2.2; R Core Team, 2022) and RStudio (version 3.0.386; Posit Team, 2023) on Windows 10 x64 (build 22621). See Supplementary Materials for more details about the R packages that were used.

Our main interest was to examine to what extent memory for individual events that constituted video clips depended on the number and duration of events that clips contained. To address this question, we focused on two indices estimating the temporal resolution of events in memory: event remembering duration and event temporal ratio. We tested our hypotheses using growth curve analyses with mixed-effect models (Mirman, 2014). We fitted two models: one with event remembering duration as outcome and one with event temporal ratio as outcome. Both models included the following predictors: a first- and second-order orthogonal polynomial transformation of event duration (i.e., linear and quadratic terms), the number of events (treated as a three-level factor) and their interaction. The first level of the “event number” factor (i.e., one event) was taken as reference level (i.e., treatment coding). Following our preregistration plan, as the conditions of application of classical linear mixed-effects models were not fully met (see https://osf.io/6aecj, for the complete assessment), we used a robust alternative (i.e., DAStau estimator; Koller, 2013, 2016; Mason, 2022)^5^. Models were fitted with the maximal random effect structure (Barr et al., 2013) and then simplified until all the parameters were properly estimated (see Supplementary Materials and osf.io/6aecj for more details). The two final models included two random effects: a random intercept for participants and a random slope for the linear term.

Finally, we fitted two additional models to examine whether the effects of event number and duration were modulated by individual differences in the vividness of visual mental imagery. The first model predicted event remembering duration by event duration, event number, VVIQ scores, and their interactions. The second model included the same predictors but event temporal ratio as outcome. In the two models, event duration was transformed in first- and second-order orthogonal polynomials (i.e., linear and quadratic terms), event number was treated as a 3-level factor, and VVIQ scores were transformed into *z*-scores. The two models included the same random effects: a random intercept for participants and a random slope (at the participant level) for the linear term.

The explained variance of the models was evaluated with Nakagawa’s R-squared (*R*^2^s). Marginal *R*^2^ represents the part of the dependent variable variance explained by fixed effects alone, while conditional *R*^2^ represents the variance explained by the entire model (fixed and random effects; Johnson, 2014; Nakagawa et al., 2017; Nakagawa & Schielzeth, 2013). We assessed the statistical significance of parameter estimates with confidence intervals (*CI*s) and *p*-values (considering an alpha of 0.05, two tailed) obtained from standard errors (*SE*) and *t*-statistics of the models using the normal approximation (i.e., treating the *t*-value as a *z*-value; Mason, 2022; Mirman, 2014). Estimated means, associated pointwise standard errors and Wald’s 95% CIs were computed based on fixed effects coefficients and variance-covariance matrices of the models.

More details about fitted models and detailed descriptive statistics are reported in the Supplementary Materials. All data, analysis scripts, and research materials are available at https://osf.io/6aecj.

### Results

#### Effects of Event Duration on Memory for Single Events.

**Figure 2.**
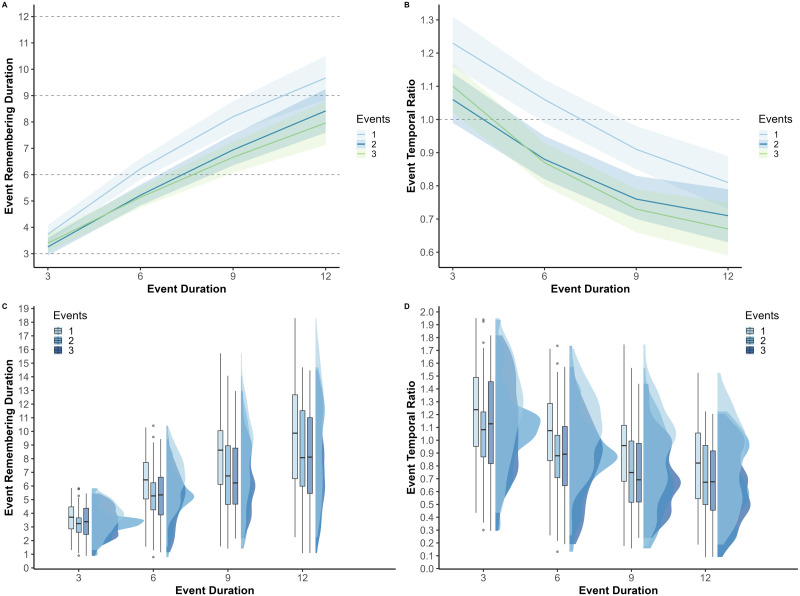
Event remembering duration and event temporal ratio as a function of event number and duration. *Note*. (**A**) Estimated event remembering duration and 95% CIs across event numbers and durations. (**B**) Estimated event temporal ratio and 95% CIs across event numbers and durations. (**C**) Observed values for event remembering duration depending on event number and duration (density and Tukey’s boxes). (**D**) Observed values for event temporal ratio depending on event number and duration (density and Tukey’s boxes).

First, we aimed to replicate previous results on the temporal limit of WM for continuous events (Leroy et al., 2024). To do so, we examined the effect of event duration on remembering duration and temporal ratio for events that were presented alone (see Figure 2). As expected, this analysis revealed a non-linear increase of event remembering duration as a function of event duration (linear term: *b* = 4.42, *SE* = 0.31, 95% CI [3.81, 5.03], *t* = 14.20, *p* < .001; quadratic term: *b* = −0.50, *SE* = 0.15, 95% CI [−0.80, −0.21], *t* = −3.35, *p* < .001). There was also a decrease of event temporal ratio as a function of event duration (linear term: *b* = −0.32, *SE* = 0.03, 95% CI [−0.38, −0.25], *t* = −9.70, *p* < .001; quadratic term: *b* = 0.04, *SE* = 0.02, 95% CI [−0.01, 0.08], *t* = 1.60, *p* = 0.11). These results show that temporal compression was not systematic but emerged when events exceeded a certain duration. According to the model estimates, event remembering duration became shorter than the actual event duration (i.e., temporal compression emerged) for events lasting 9 s or longer (see Table 1).

**Table 1.**
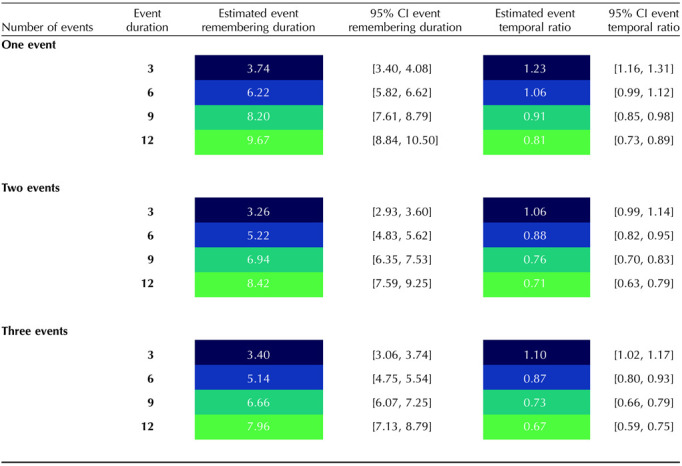
Estimated event remembering duration and event temporal ratio as a function of event number and duration.

#### Effects of Event Number and Duration on Memory for Events Presented in Sequence.

Next, we investigated whether, in addition to being impacted by event duration, the temporal resolution of events in memory decreases when they are encoded as part of a broader sequence of events. There was indeed a significant effect of the number of events on the index of event remembering duration (see Figure 2A), showing that remembering duration was higher when events were presented in isolation than when they were included in videos that contained several events (one event vs. two events: *b* = 1.00, 95% CI [0.74, 1.25], *t* = 9.44, *p* < 0.001; one event vs. three events: *b* = 1.17, 95% CI [0.91, 1.42], *t* = 11.04, *p* < 0.001); remembering duration did not differ significantly depending on whether two or three events had to be mentally replayed (*b* = 0.17, 95% CI [−0.08, 0.42], *t* = 1.65, *p* = 0.32). A similar pattern of results was obtained for the event temporal ratio (see Figure 2B): the ratio was lower when several events were presented (one event vs. two events: *b* = 0.15, 95% CI [0.11, 0.18], *t* = 9.42, *p* < 0.001; one event vs. three events: *b* = 0.16, 95% CI [0.13, 0.20], *t* = 10.48, *p* < 0.001) but did not differ significantly depending on whether two or three events had to be mentally replayed (*b* = 0.02, 95% CI [−0.02, 0.05], *t* = 1.10, *p* = 0.86).

We also examined to what extent the effect of event duration on temporal compression varied depending on the number of events included in the sequence. We expected the number of events to have a stronger effect for longer events, as these contain more redundant information that can be omitted. In other words, we predicted that the increase of event remembering duration with event duration would be attenuated when events are presented in a sequence. In line with this hypothesis, there was a significant interaction between the number of events and the linear term for event duration, showing that the increase in remembering duration with event duration was smaller (the effect of the linear term was less pronounced) when events were presented in sequences of two and three events than when they were presented alone (two events vs. one event: *b* = −0.58, 95% CI [−0.99, −0.16], *t* = −2.73, *p* = 0.01; three events vs. one event: *b* = −1.02, 95% CI [−1.43, −0.61], *t* = −4.83, *p* < 0.001). Regarding event temporal ratio, no significant interaction was found (two events vs. one event: *b* = 0.05, 95% CI [−0.01, 0.12], *t* = 1.73, *p* = 0.084; three events vs. one event: *b* = −0.002, 95% CI [−0.06, 0.06], *t* = −0.06, *p* = 0.96). We hypothesized that, when presented within a sequence, events would be chunked (represented more synthetically, in a compressed form) to allow them to be maintained in WM while processing subsequent events. Following this view, the constant impact of the number of events on the temporal ratio, regardless of event duration, suggest that this WM compression of continuous events is accomplished by omitting a fixed proportion of the event unfolding.

#### Additional Analyses on the Effects of Event Number and Duration.

To further characterize the effects of the number and duration of events on the temporal compression of memories, we conducted some additional (non-registered) analyses. These are reported in detail in the Supplementary Materials and the main results are summarized below. As detailed above, we found that events were more compressed in memory when they were part of a sequence that included several events. However, it is worth noting that, despite this decrease in event remembering duration, mental replay times for the entire video clips (i.e., for the sequence of events) increased with the number of events they contained (i.e., participants took longer to remember videos that included more events). We also examined whether, for a given video duration, the video temporal ratio (i.e., the ratio between the time taken to remember the entire sequence of events and its actual duration) varied according to the number of events it contained. This showed that, for the same video duration, the video temporal ratio was higher (i.e., temporal compression was lower) when the video contained more events. These results are consistent with previous studies showing that past episodes are remembered with less temporal compression when they include more event boundaries (i.e., more events; Folville et al., 2020; Jeunehomme et al., 2020; Leroy et al., 2025; Wang & Gennari, 2019).

To ensure that the effects of event number and duration reported above were not dependent on our choice of statistical model (growth curve modeling with orthogonal linear and quadratic terms), we reanalyzed the data using an alternative approach that does not impose a specific functional form (e.g., quadratic) on the relationship between predictors and outcome. Specifically, we used a Generalized Additive Mixed Model (GAMM), which enables flexible, data-driven modeling of nonlinear relationships using smooth functions, such as splines (Pedersen et al., 2019). Rather than imposing a predefined functional form, these models identify the shape of the relationship that best fits the data. To prevent excessively wiggly models and control for overfitting, a ‘wiggliness’ penalty is applied to the model fitting objective. This penalty depends on the model’s complexity: the more complex the model, the higher the penalty.

Using the mgcv package in R (Wood, 2023), we fitted a GAMM predicting event remembering duration by event duration (smoothed term—we used the mgcv’s default low-rank thin plate spline), event number (treated as a three-level ordered factor) and their interaction. Following recommendations of Pedersen et al. (2019), model coefficients were estimated using restricted maximum likelihood (REML). The model included a random intercept for participants and a random slope for the effect of event duration. We obtained the same pattern of results as the one reported above with the growth curve approach. First, we examined the effect of event duration on remembering duration for events that were presented alone. As expected, this analysis revealed a non-linear increase of event remembering duration as a function of event duration (EDF = 2.43, *F* = 102.18, *p* < .001). The EDF of 2.43 indicates mild nonlinearity, with a smooth curve that approximates a quadratic relationship (a curve with one bend), as a quadratic function typically corresponds to an EDF of 2.^6^ There was also a significant effect of the number of events on the index of event remembering duration, showing that remembering duration was higher when events were presented in isolation than when they were included in videos that contained several events (two events vs. one event: estimate = −1.02, 95% CI [−1.27, −0.78]; three events vs. one event: estimate = −1.23, 95% CI [−1.47, −0.99]); remembering duration did not differ significantly depending on whether two or three events had to be mentally replayed (estimate = 0.21, 95% CI [−0.03, 0.45]). Finally, there was a significant interaction between the number of events and event duration, showing that the increase in remembering duration with event duration was smaller when events were presented in sequences of two and three events than when they were presented alone (one vs. two: EDF = 1, *F* = 5.08, *p* = 0.03; one vs. three: EDF = 1, *F* = 18.89, *p* < .001).

We also tested the possibility that the temporal resolution of event representations in memory may primarily be determined by the total duration of the sequence in which they are included, rather than by the number and duration of events as such. To investigate this possibility, we compared the goodness of fit (using seven indices) of the models we fitted to predict event remembering duration and event temporal ratio, on the one hand, and models predicting the same outcome simply by the total video duration, on the other hand. For each of the indices, the model with event number and duration as predictors outperformed the model with the total video duration as single predictor, both for event remembering duration and event temporal ratio (see Supplementary Materials).

#### Individual Differences in Visual Imagery.

**Figure 3.**
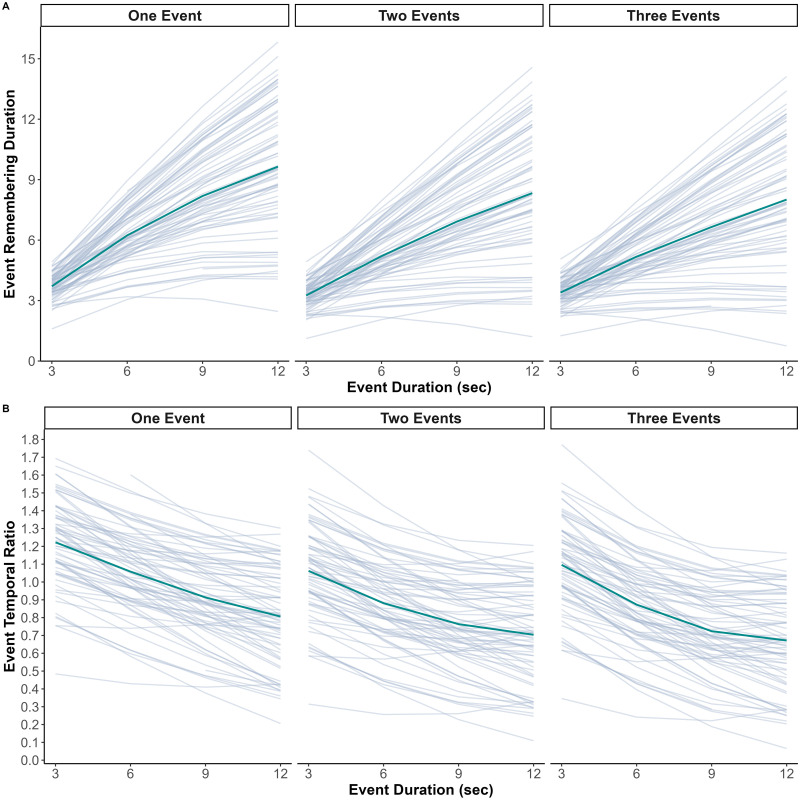
Individual differences in event remembering duration and temporal ratio as a function of event number and duration. *Note*. Fitted value from the two robust linear mixed-effects models we fitted to test our main hypothesis (see Statistical Analyses section). Each grey line represents fitted values of a participant. The green line represents the global trend according to the model estimates. (**A**) Event remembering duration as a function of event number and duration. (**B**) Event temporal ratio as a function of event number and duration.

Our data revealed substantial individual differences in memory compression rates (see Figure 3). Although this was not the primary objective of our study, we explored factors that could explain these individual differences. More specifically, we examined to what extent participants’ event remembering duration and event temporal ratio, as well as the effects of event number and duration, were related to individual differences in visual imagery.

We expected to observe a positive association between VVIQ scores and event remembering duration and temporal ratio. Moreover, we expected that individuals with high vividness of visual imagery would be less subject to the deleterious effects of event number and duration. The results partially supported our hypotheses (see Figure S8). There was no significant main effect of VVIQ scores on event remembering duration (*b* = 0.17, 95% CI [−0.28, 0.61], *t* = 0.73, *p* = 0.468) or event temporal ratio (*b* = 0.01, 95% CI [−0.05, 0.07], *t* = 0.35, *p* = 0.727), but there were significant interactions between VVIQ scores and the effect of event duration. Indeed, the increase of event remembering duration with event duration was positively associated with VVIQ scores (interaction between the linear term and VVIQ scores: *b* = 0.50, 95% CI [−0.05, 1.05], *t* = 1.78, *p* = 0.075; interaction between the quadratic term and VVIQ scores: *b* = 0.24, 95% CI [0.07, 0.41], *t* = 2.77, *p* = 0.006). Furthermore, participants with high VVIQ scores showed a weaker decrease of event temporal ratio with event duration (interaction between the linear term and VVIQ scores: *b* = 0.05, 95% CI [0.00, 0.11], *t* = 2.07, *p* = 0.038, interaction between the quadratic term and VVIQ scores: *b* = 0.01, 95% CI [−0.01, 0.04], *t* = 0.94, *p* = 0.346). These results suggest that the increase in temporal compression of memories with event duration is lower for people who have a higher visual imagery capacity. In contrast, there was no significant interaction between the effect of the number of events and VVIQ scores, neither for event remembering duration (two vs. one event: *b* = 0.11, 95% CI [−0.10, 0.31], *t* = 1.03, *p* = 0.302, three vs. one event: *b* = 0.13, 95% CI [−0.08, 0.34], *t* = 1.21, *p* = 0.226), nor for event temporal ratio (two vs. one event: *b* = 0.01, 95% CI [−0.02, 0.04], *t* = 0.74, *p* = 0.458, three vs. one event: *b* = 0.02, 95% CI [−0.01, 0.05], *t* = 1.00, *p* = 0.317).

### Discussion

Using a mental replay task with video clips in which the number and duration of events varied orthogonally, we showed that both dimensions influenced the temporal resolution of event memories. In line with previous findings (Leroy et al., 2024), we observed that the temporal compression of single continuous events emerged when they lasted 9 s or longer. We then found that events were mentally replayed more quickly when they were part of a sequence of several events (i.e., two or three events) than when they were presented alone, particularly for long events. In contrast, the difference between sequences of two and three events was small and not statistically significant. Further analyses revealed that the temporal resolution with which events composing a sequence were represented in memory was better explained by the microstructure of the sequence (i.e., event number and duration) than by its total duration. Finally, we observed that the increase in event remembering duration with event duration was greater for participants with higher vividness of mental imagery. Coherently, these individuals showed a less pronounced decrease in event temporal ratio with event duration. On the other hand, we did not observe any significant interaction between the vividness of mental imagery and the effect of event number. Taken together, the results of Experiment 1 provide initial evidence that event number and duration jointly shape the temporal resolution of memories.

## EXPERIMENT 2

A limitation of Experiment 1 is that event remembering duration was estimated indirectly by dividing the total replay time of an entire video by the number of events it contained. To address this, Experiment 2 was designed to replicate the main findings of Experiment 1—the effects of event number and duration on event remembering duration—while directly measuring remembering duration for each individual event presented in the videos. Participants completed a similar event memory task as in Experiment 1, but when videos contained multiple events, they pressed a key during mental replay whenever they transitioned from one event to the next. This adjustment enabled us to measure replay duration for each individual event, rather than estimating it from total video replay time. This experiment was not preregistered. Data, analysis scripts, and materials are available at osf.io/6aecj.

### Methods

#### Participants.

Seventy-two young adults aged between 18 and 35 years (*M* = 29.03, *SD* = 4.99; 33 women, 35 men, 4 undefined) were recruited on Prolific (https://www.prolific.co/; Peer et al., 2022) and received £2 compensation for their participation (average duration: 15 min). The sample size was chosen to match that of Experiment 1. All participants were native English speakers from the USA and UK and, to be eligible, they had not to be currently taking any medication that could affect their ability to concentrate or have a history of psychiatric, psychological, or neurological disorders. The study was approved by the local ethics committee (ref. 2122-009).

#### Materials and Procedure.

**Figure 4.**
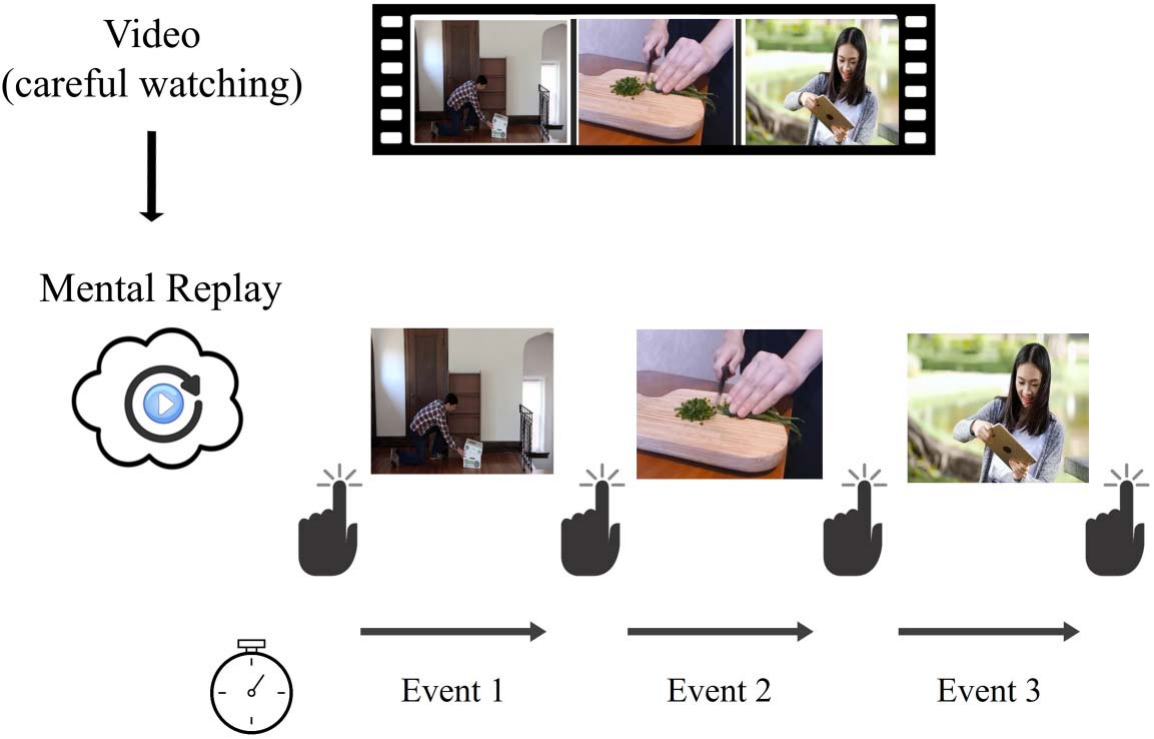
Illustration of a trial in the mental replay task used in Experiment 2. *Note*. Participants had to watch and mentally replay short videos depicting 1, 2, or 3 continuous events (i.e., without event boundary), each lasting 3, 6, 9, or 12 s. For videos containing multiple events, they pressed a key each time they transitioned from one event to the next during mental replay. The intervals between successive keypresses were taken as proxies for the mental replay duration of the corresponding events. For example, in a three-event video, the time between the first and second keypresses reflected the replay duration of the first event, the time between the second and third keypresses reflected the remembering duration of the second event, and the time between the third and the fourth keypresses represented the mental replay duration of the last (third) event.

Participants completed a similar event memory task as in Experiment 1, using the same stimuli. The only difference was that, for videos containing several events, they were instructed to press a key each time they transitioned from one event to the next during mental replay (see Figure 4). This procedure was adapted from Herbst et al. (2025). On each trial, participants performed two, three, or four keypresses depending on the number of events depicted in the video (one, two, or three). Specifically, when the video contained only one event, they pressed the key once when they began mentally replaying the event and once when they finished. When a video contained two events, they pressed the key once when they started remembering the first event, once when transitioning from the first to the second event, and once when they finished mentally replaying the second event. When a video contained three events, they pressed the key once when they started remembering the first event, once when transitioning from the first to the second event, once when transitioning from the second to the third event, and once when they finished mentally replaying the third event. The intervals between successive keypresses were taken as proxies for the mental replay duration of the corresponding events (see Figure 4).

Before starting the experimental trials, participants received written instructions and watched a video tutorial (filmed from a first-person perspective) summarizing the procedure and how to perform the task properly. The instructions and video tutorial are publicly available on OSF (osf.io/6aecj).

#### Data Cleaning and Statistical Analyses.

With the procedure employed in Experiment 2, we obtained one, two, or three observations—remembering durations—per trial, depending on the number of events depicted in the video. The same exclusion criteria as in Experiment 1 were applied: observations were excluded if remembering duration was shorter than 1 s (to account for accidental double keypresses) or longer than twice the actual duration of the remembered event (to account for interruptions or cases where the event was not mentally replayed in one shot). For multi-event trials, the entire trial was discarded whenever one remembering duration did not meet these criteria. We also excluded trials in which participants took more than 15 s to begin their mental replay of the sequence, as well as trials affected by technical issues in video presentation (e.g., an abnormal duration of presentation). The entire participant was excluded if more than half of their trials for a given event duration or event number had to be removed. Each excluded participant was replaced by another. In total, 20 participants were excluded. The final analyses are based on 1,665 observations from 72 participants. Raw (uncleaned) data are available at osf.io/6aecj.

**Figure 5.**
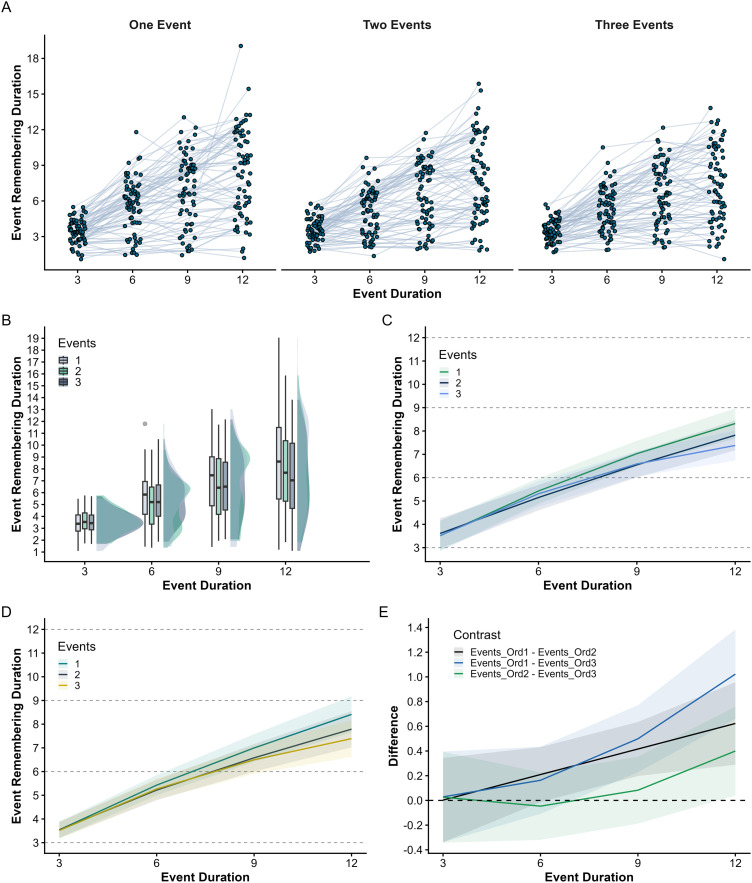
Overview of the data and the model fitted values in Experiment 2. *Note*. (**A**) Observed values for event remembering duration (aggregated at the trial level). Data points from the same participant are linked by a grey line. (**B**) Distribution of event remembering duration across event number and duration (density and Tukey’s boxes). (**C**) Estimated event remembering duration and 95% CIs across event numbers and durations (growth curve model). (**D**) Estimated event remembering duration and 95% CIs across event numbers and durations (GAMM). (**E**) Estimated differences in remembering duration between event numbers across event durations (derived from GAMM).

When videos contained multiple events, the remembering durations of individual events were averaged to yield a single value per trial. This allowed us to fit the same growth curve model as in Experiment 1, examining the extent to which memory for individual events within video clips depended on the number and duration of the events. The model predicted event remembering duration from a first- and second-order orthogonal polynomial transformation of event duration (i.e., linear and quadratic terms), the number of events (treated as a three-level factor), and their interaction. As in Experiment 1, we verified the robustness of our findings by reanalyzing the data with a GAMM, thereby ensuring that the results did not depend on the specific modeling approach. For both analyses, the model specification, predictor coding, and inferential tests were identical to those used in Experiment 1. Observed event remembering durations are displayed in Figure 5 (panels A and B). Detailed descriptive statistics can be found at osf.io/6aecj.

### Results

#### Effects of Event Duration on Memory for Single Events.

We first examined the effect of event duration on remembering duration for events presented alone. As in Experiment 1, remembering duration increased non-linearly with event duration (linear term: *b* = 3.59, *SE* = 0.28, 95% CI [3.03, 4.14], *t* = 12.68, *p* < .001; quadratic term: *b* = −0.31, *SE* = 0.12, 95% CI [−0.55, −0.08], *t* = −2.61, *p* = 0.01). Event remembering duration was close to the actual event duration for short events but became shorter (i.e., temporal compression emerged) as events increased in length (Figure 5C).^7^ This finding was corroborated by a GAMM, which revealed a non-linear effect of event duration on remembering duration (EDF = 2.3, *F* = 82.52, *p* < .001; see Figure 5D). The EDF indicates mild nonlinearity, with a smooth curve that approximates a quadratic relationship (a curve with one bend).

#### Effects of Event Number and Duration on Memory for Events Presented in Sequence.

We next tested whether the temporal resolution of event memory decreased when events were part of a sequence. There was a significant effect of event number on remembering duration (Figure 5): remembering durations were longer when events were presented in isolation than when they were embedded in sequences of two or three events (one event vs. two events: *b* = 0.29, 95% CI [0.88, 0.5]; one event vs. three events: *b* = 0.37, 95% CI [0.17, 0.58]). The difference between two- and three-event sequences was smaller and not significant (*b* = 0.08, 95% CI [−0.12, 0.29]). The GAMM analysis yielded a similar pattern: remembering duration was higher for single events than for events in two- or three-event sequences (two events vs. one event: *b* = −0.32, 95% CI [−0.52, −0.12]; three events vs. one event: *b* = −0.43, 95% CI [−0.64, −0.23]).

Finally, both modeling approaches revealed an interaction between event duration and event number, showing that the increase in remembering duration with event duration was attenuated when events were presented within sequences (growth curve, one vs. two: *b* = 0.45, 95% CI [0.04, 0.86], one vs. three: *b* = 0.71, 95% CI [0.30, 1.12]; GAMM, one vs. two: EDF = 1, *F* = 5.03, *p* = 0.03, one vs. three: EDF = 1.8, *F* = 7.01, *p* < .001). In other words, the effect of the number of events grew stronger as the duration of the events increased (see Figure 5E).

### Discussion

Experiment 2 replicated the key findings of Experiment 1: temporal compression appeared once events exceeded a certain duration, and events were mentally replayed more quickly when part of a sequence of two or three events than when presented alone, particularly for longer events. Importantly, this was demonstrated when remembering duration for each event was measured directly, rather than inferred from the total replay duration of entire sequences as in Experiment 1. Together, these results provide converging evidence that both event number and event duration constrain the temporal resolution of event memory.

## GENERAL DISCUSSION

The current study aimed to shed light on the mechanisms underlying the construction of event sequences in memory by inspecting how the microstructure of the perceptual stream shapes the temporal compression of events in memory representations. Across two experiments, we showed that the number and duration of events both influence the temporal resolution of event memories. In line with previous findings (Leroy et al., 2024), we observed that the temporal compression of single continuous events is not systematic but emerges when they exceed a certain duration. We then found that events were mentally replayed more quickly when they were part of a sequence of several events (i.e., two or three events) than when they were presented alone, particularly for long events. In contrast, the difference between sequences of two and three events was small and not statistically significant.

According to seminal models of event cognition (Loschky et al., 2020; Zacks, 2020), to make sense of “what is happening now”, we continually construct a mental model of the current situation—an event model that contains information about people, objects, actions, and perceptual features of the situation. This model is maintained in WM and accumulates information representing the unfolding of experience until a coarse event boundary is perceived, which triggers the transfer of the event model to long-term memory (i.e., as an experience unit or a sequence of experience units; Baldassano et al., 2017; Lu et al., 2022). Given the limited capacity of WM, at least two characteristics of the stream of events may influence this encoding mechanism, thereby shaping the temporal compression of events in memory representations. First, when the duration of a continuous event that needs to be maintained exceeds WM capacity, the mental representation of its unfolding may be truncated, leading to an incomplete encoding of within-event information in long-term memory. In support of this view, and replicating previous results (Leroy et al., 2024), we found that the mental replay of a continuous event begins to be shorter than the actual event duration when it exceeds a certain duration. Second, the temporal resolution of event representations may be further diminished when several events need to be retained. When experiencing a sequence of events, fine event boundaries may induce the chunking of information that needs to be maintained in WM: each boundary may trigger the creation of a WM representation that represents the unfolding of the just-experienced event in a compressed form, freeing up WM resources for the maintenance of subsequent events. This chunking mechanism may reduce the precision of memory representations, which may in part explain the time-compressed replay of events that are maintained in WM. In support of this view, we found that, beyond event duration, the memory replay of an event is more compressed when it is part of a sequence of several events than when it is presented alone. Moreover, we observed a stronger effect of event number on remembering duration for longer events, suggesting that maintaining several events in memory comes at the expense of some within-event information. This result echoes those of previous studies that have shown that temporal discontinuities in memory representations correspond to moments of past experience that occurred between event boundaries (i.e., within-event information; Jeunehomme & D’Argembeau, 2020, 2023; Michelmann et al., 2023).

Although the role of chunking in optimizing the maintenance of information in WM has been abundantly demonstrated (for a review, see Norris & Kalm, 2021), most studies involve stimuli that lack the perceptual richness and temporally extended nature of real-world events. The present study thus provides preliminary evidence for the role of chunking in representing dynamic, naturalistic events in WM. Our results suggest that the chunking mechanism observed in classical WM tasks (i.e., the recoding of redundant information into a more synthetic representation) may be involved in the construction of event models. Indeed, it has been suggested that event segmentation could be seen as a temporal chunking of the perceptual stream, leading to the formation of compressed event representations (Clewett & Davachi, 2017; Clewett et al., 2019). However, this proposal was based on studies in which events consisted of sequences of static stimuli (e.g., pictures) sharing the same context (e.g., pictures of people vs. pictures of objects). Our study therefore goes a step further, suggesting that a similar chunking mechanism of the perceptual stream could be (at least partly) responsible for the temporal compression of naturalistic events in memory.

In Experiment 1, we found that the increase of memory compression rates with event duration was less pronounced for people who have more vivid visual imagery. This result is notable from both a methodological and theoretical perspective. Methodologically, it supports the validity of our mental replay time measure as a reflection of actual replay duration, rather than the time needed to search information in memory (which could also vary according to the number and duration of events). This interpretation is indeed consistent with evidence that individuals with better visual imagery recall events in greater detail (Marks, 1973; Sheldon et al., 2017; Vannucci et al., 2016), and thus in a less compressed form. From a theoretical standpoint, our results align with the view that the ability to form visual mental images and the ability to integrate the unfolding of continuous events both rely on WM. There is substantial evidence of overlap between visual mental imagery and visual WM, as both involve the active maintenance and manipulation of visual information (Brockmole, 2008; Tong, 2013) and rely on a depictive representational format (Borst et al., 2012). This overlap is supported by both behavioral and neuroimaging studies (Albers et al., 2013; Baddeley & Andrade, 2000; Ceja & Franconeri, 2023; Keogh & Pearson, 2011). In addition, previous studies suggest that the length of continuous events that can be held entirely in WM varies depending on individuals’ WM capacity (Leroy et al., 2025). In line with these previous findings, our results suggest that the temporal resolution with which events are represented in memory may depend on the ability to maintain/accumulate visual information in WM. Conversely, the effect of the number of events on compression did not significantly vary with individuals’ mental imagery, suggesting that event number and duration influence the temporal resolution of memory through distinct mechanisms.

While the present results suggest that event segmentation and WM capacity jointly determine the temporal structure of everyday memories, several questions remain open. In line with the event chunking mechanism we proposed, our results suggest that the unfolding of events is encoded less completely when they are experienced in a sequence. However, since we did not assess the specific content of mental replay, our data do not permit precise conclusions about how this chunking process is implemented—that is, how events are recoded in memory. The observation that event remembering duration increased with event duration, even when several events had to be retained, suggests that within-event information is not entirely dropped during chunking. The removal of redundancies is thought to be one of the key mechanisms by which information is compressed in memory (Bates & Jacobs, 2020; Norris & Kalm, 2021). Although this remains to be empirically tested, the chunking/compression of event representations in WM could be achieved by retaining only some instances of redundant perceptual elements. For example, repetitions of the same action (e.g., the different turns made by a person unscrewing a bolt) could be discarded to form more efficient representations.

Based on previous research showing the precision of WM representations declines as the number of items increases (Bays et al., 2009; Bays & Husain, 2008; Hepner & Nozari, 2019; Joseph et al., 2015; Tsuda & Saiki, 2019; Zokaei et al., 2011), we expected the temporal resolution of event representations to decrease with their number. However, we found no significant difference in event temporal resolution between sequences composed of two versus three events. To further clarify the effect of the number of events on the temporal resolution of memory representations, future studies should examine sequences containing more than three events. It might also be that the temporal resolution of event representations depends not on the number of events per se, but on the number of layers in the segmental structure of the sequence. In our study, since the events that composed the sequences were unrelated, we had only two types of segmental structures: videos showing a single event (one layer) and videos showing sequences of several unrelated events (two layers: each individual event and the sequence they form together). It could be that the temporal compression of events is more sensitive to the number of layers in an event sequence than the number of events per se, which could explain why there was no significant difference between sequences of two versus three events. Future research should aim to disentangle the respective contributions of event number and hierarchical structure to temporal compression in memory^8^.

According to our view, WM maintains and integrates fine-grained events by accumulating them until a coarse event boundary is encountered. At that point, all the accumulated fine events are collectively transferred to episodic memory as a sequence of experience units. Temporal compression would thus result from WM capacity limit in accumulating long and/or numerous events during online perception. While our results are consistent with this mechanism, alternative cognitive processes could underlie the effects of event number and duration on temporal compression. One possibility would be that every event boundary, even those delimiting low-level or fine-grained events, triggers the transfer of the current event model to long-term memory. Then, when a coarse event boundary is perceived, these previously stored events would be reactivated in WM and re-encoded as a meaningful unit in long-term memory (Güler et al., 2024). Under this account, the temporal resolution with which events are represented in episodic memory would be essentially determined during this re-encoding process. More precisely, the quantity of perceptual information that can be reactivated “in one shot” in WM could be limited, such that the temporal resolution with which events are reactivated would decrease with event number and duration (leading to missing segments in the re-encoded experience unit). Such a mechanism could also account for the pattern of results observed in the current study.

While the present results suggest that the microstructure of the perceptual stream shapes the encoding of events—leading to more or less compressed memory representations—the partial loss of within-event information when representing a sequence of events may also stem from processes operating at retrieval. Recent research has shown that, when asked to mentally replay past episodes, people tend to recall the most informative parts of the episode (typically those surrounding event boundaries) while skipping less relevant, often redundant, within-event details (Michelmann et al., 2019, 2023). In line with these results, it has been suggested that the speed of event simulations can be adaptatively modulated to maximize the trade-off between simulation accuracy and duration (Arnold et al., 2016). In this context, the speed of mental replay of within-event information may increase with the number of remembered events. That is, when several events have to be remembered, mental replay may be speeded up between event boundaries by leaving out redundancies. As a result, temporal compression would be higher for episodes composed of long and numerous events. Therefore, temporal compression may reflect not only encoding constraints but also adaptive retrieval strategies.

One may also wonder to what extent the present findings relate to previous research on memory for durations (Bangert et al., 2020; Herbst et al., 2025; Lejeune & Wearden, 2009; Wang & Gennari, 2019). Although distinct, the mental replay of an event and the estimation of its duration may partially draw upon shared cognitive processes^9^. The estimation of a past event’s duration often relies on the memory representation of what occurred during the interval—i.e., on non-temporal information (Block & Reed, 1978; Block & Zakay, 1997). In line with this view, events (of the same length) are judged as longer when they contain more event boundaries (Faber & Gennari, 2015; Roseboom et al., 2022; Wang & Gennari, 2019), likely due to weaker encoding of within-event information (Wang & Gennari, 2019). Thus, memory for events and memory for durations partly rely on the same (sometimes incomplete) source of information (i.e., past event models). However, duration memory may also involve additional, time-specific processes. Memory for the duration of intervals that do not include perceptual changes (e.g., “empty” intervals delimited by two auditory tones) is thought to involve the memorization of temporal information itself (Block & Zakay, 1997). Seminal theories proposed the idea of a mental pacemaker, which produces pulses at a particular rate according to the organism’s arousal. Pulses captured by attention would be accumulated in WM to form mental representations of duration. From this perspective, the accuracy of duration memory depends on specific processes that are not necessarily engaged in the mental replay of everyday events (such as the allocation of attentional resources to the passage of time; Allman et al., 2014; Dutke, 2005; Gibbon et al., 1984).

Another issue that warrants further discussion is our finding that remembering durations for short events (i.e., 3-s events) were slightly longer than the actual event durations (temporal ratio > 1). This result was unexpected, and one possible explanation is the presence of a central tendency bias—a well-known phenomenon in which judgments are biased toward the average of previously encountered values.^10^ To investigate this possibility, a recent study conducted in our lab (Leroy & D’Argembeau, 2025) systematically manipulated the duration of the shortest events (3, 6, 9, or 12 seconds) presented within a set of stimuli. The goal was to determine whether the longer mental replay durations for short events could be attributed to such a bias. Four groups of participants viewed and then mentally replayed videos depicting single continuous events, similar to those used in the current study. The range of event durations varied by group: group 1 saw 3-, 6-, and 9-s videos; group 2 saw 6-, 9-, and 12-s videos; group 3 saw 9-, 12-, and 15-s videos; and group 4 saw 12-, 15-, and 18-s videos. If mental replay durations were solely driven by a central tendency bias, the shortest events should be replayed with durations longer than their actual lengths in all groups. However, this was not consistently observed. Replay durations exceeded actual durations for 3-s events and 6-s events, but not for longer ones: 9-s events were replayed with durations closely matching their actual lengths, and 12-s events were replayed more quickly than they were originally experienced. These findings suggest that a central tendency bias alone cannot fully account for the pattern of results observed in the current study. Indeed, it appears that beyond a certain duration (around 9 s), continuous events tend to undergo temporal compression in memory, even when presented alongside longer events. This suggests that mechanisms other than central tendency bias, such as memory-based compression processes, contribute to the shaping of mental replay durations. In addition, it should be reminded that, in the current study, each participant saw each possible combination of event number and duration only once, resulting in a total of 12 unique stimuli. It therefore seems unlikely that participants based their response on knowledge of the statistical distribution of stimuli rather than on their memory of the event they had just seen.

An alternative interpretation of the finding that the shortest events were mentally replayed for longer than their actual duration concerns the nature of our measure of remembering duration. Specifically, the time participants took to indicate the beginning and end of their mental replay likely reflects the combined durations of two distinct cognitive processes: the time needed to mentally replay the unfolding of the event (our process of interest) and the time taken to access the initial memory trace (i.e., the time needed to represent the initial visual scene from which the participants had to mentally replay the subsequent unfolding of the event). When this retrieval initiation phase is taken into account, the total measured duration will inevitably exceed the original event length in cases where the replay unfolds at a natural (i.e., non-compressed) pace. In this light, the observed temporal ratios slightly above 1 for short events may reflect the additional time required to initiate the mental replay. Supporting this interpretation, a recent study found that when remembering duration estimates were corrected for retrieval initiation time—assessed in a separate task—temporal ratios no longer exceeded 1, even for the shortest (i.e., 3-s) events (Leroy & D’Argembeau, 2025).

Beyond the microstructure of events, some features of the individual events that compose a sequence (e.g., their sensorial richness or familiarity) could modulate the effect of duration on memory compression. In the current study, as we were specifically interested in the effects of event number and duration, the potential influences of these other features were controlled through counterbalancing of stimuli. Nevertheless, identifying the various characteristics of events shaping their temporal resolution in memory is an important avenue for future research (see Colson & D’Argembeau, 2025). In addition, the position of the event in the sequence could also influence the temporal resolution with which it is encoded in memory. In this study, the effect of position was controlled by counterbalancing, but it would be interesting in future research to examine whether primacy and recency effects are apparent in the temporal resolution with which events are mentally replayed.

In conclusion, the current study sheds a new light on how the microstructure of the perceptual stream shapes its temporal resolution in memory. We showed that the temporal resolution of memory representations depends on both the number and duration of events composing past episodes. More precisely, our findings suggest that the temporal compression of events may result from WM capacity limit in representing continuous events and the chunking of within-event information. These results mark a step forward in the study of the temporal compression of events in episodic memory, this pervasive but poorly understood aspect of naturalistic event representation.

## ACKNOWLEDGMENTS

We thank Olivier Jeunehomme for his help throughout the realization of this study.

## FUNDING INFORMATION

This work was supported by ULiège with a grant “Crédits et Projets de Recherche en Sciences Humaines 2024” (FSR-S-SH-PDR-24/12) and a grant from the Wallonia-Brussels Federation – Concerted Research Actions (ARC 23/27-05 – COMPRESS). Nathan Leroy and Arnaud D’Argembeau are, respectively, Research Fellow and Research Director at the Fonds de la Recherche Scientifique (F.R.S.-FNRS), Belgium.

## AUTHOR CONTRIBUTIONS

Nathan Leroy: Conceptualization, Formal analysis, Investigation, Methodology, Writing – original draft. Arnaud D’Argembeau: Conceptualization, Methodology, Supervision, Writing – original draft.

## DATA AVAILABILITY STATEMENT

The design of this study, the analysis plan, and all hypotheses were preregistered on OSF (https://osf.io/a6ydj). We report how we determined our sample size, all data exclusions, transformations, and all measures. All data, analysis scripts, and research materials are available at https://osf.io/6aecj.

## Notes

^1^ Research on temporal cognition suggests that the “subjective present” lasts about 3 s, which would correspond to the elementary units of the flow of consciousness (Fairhall et al., 2014; Monfort et al., 2020; Montemayor & Wittmann, 2014; Pöppel, 1997). Working memory would then enable us to maintain several of these units of “now” in an active state to form more complex event representations (i.e., event models; Richmond & Zacks, 2017) covering up to 10–12 s of the perceptual stream (Jeneson & Squire, 2012; Wittmann, 2016). Based on this literature, we decided to sample continuous events lasting from 3 to 12-s, in 3-s increments.^2^ The transition between different events in a sequence was made by a relatively fast fading: each event started with a black frame, followed by 8 frames during which the activity gradually appeared. Similarly, at the end of each event, the activity gradually disappeared for 8 frames, ending with a black frame. To avoid introducing any systematic visual differences between events presented alone or in a sequence, events began and ended in this way even when presented alone. For all our video stimuli, the frame rate was 30 fps.^3^ During the pilot study, participants were asked to write down the content of their memory immediately after each stimulus had been mentally replayed. The number of recalled events corresponded to the number of events presented in the video on nearly all trials. Of note, some participants reported being distracted during the viewing and mental replay of the video because they were wondering which words they would use to describe the events (e.g., one participant reported that “during the video with the man changing a tire, I kept asking myself the name of the tool used because I knew I would have to describe the scene”). Therefore, to simplify the task and maximize the involvement of participants in the mental replay of the videos, we decided to remove the written description part of the task in the main study.^4^ As suggested by McKelvie (1995), the numerical values on the 5-point rating scale initially proposed by Marks were reversed, so that higher ratings represent greater vividness.^5^ The results obtained with the classical and robust analyses were similar and conclusions regarding our hypotheses were the same. We therefore only report here the results obtained with the robust estimates, while the results of the classical analyses are available at https://osf.io/6aecj.^6^ One critical concept for interpreting GAMMs is the Effective Degrees of Freedoms (EDF), which reflects the degree of non-linearity of a curve. An EDF value close to 1 indicates that the relationship between the outcome and the smooth term is approximately linear, whereas higher EDF values suggest that the relationship modeled is more complex and nonlinear (in a way, the EDF represents the degree of non-linearity of the relation; Wood, 2017).^7^ Despite a similar pattern of results, remembering duration for single events was shorter overall in Experiment 2 compared to Experiment 1, and temporal compression emerged at shorter event durations (see Figure 5). We do not have a clear explanation for these differences, but they may result from the less controlled environment of online data collection, which may have introduced noise or careless responses.^8^ This could be investigated with stimuli varying in the complexity of their hierarchical structure. For example, a sequence could include four events that are unrelated (two hierarchical layers) or could be composed of two events from the same theme and two other events from another theme (e.g., first event: someone mows the lawn, second event: the same person cuts the hedge, third event: another person runs in town, fourth event: the same person runs in the woods). The latter sequence has a 3-layer hierarchical structure: each individual event, the two events from the same theme, and the entire sequence.^9^ This could explain the similarity between the decrease of temporal ratio with event duration observed in the current study and the decrease of the “estimated duration/actual duration” ratio typically observed with tasks assessing memory for durations (e.g., Faber & Gennari, 2015; Gümüş & Balcı, 2023; Herbst et al., 2025; Roseboom et al., 2022; Wang & Gennari, 2019).^10^ In the context of time perception, this bias is closely related to Vierordt’s law, which posits that, when estimating a range of time intervals, individuals tend to overestimate short durations and underestimate longer ones, with an “indifference point” in-between—more accurate judgments occurring for intermediate durations (Lejeune & Wearden, 2009; Shi et al., 2013).

## Supplementary Material


