# Supplementary material for: The Role of Event Number and Duration in Time-Compressed Memory Replay
Source: Open Mind (Camb). 2025 Dec 18;9:2066–91. doi: 10.1162/OPMI.a.276 (PMC12768557; doi:10.1162/OPMI.a.276)
Supplement: Supplementary file 1 [file opmi-09-2066-s001.pdf]

Supplementary Materials for Experiment 1

Supplementary information about statistical analyses

Descriptive statistics

The Results section of the main text focuses on estimates from the robust linear mixed-effects models. For the sake of completeness, here we provide more detailed descriptive statistics about our main outcome variables: event remembering duration (Table S1) and event temporal ratio (Table S2).

Table S1

Event remembering duration as a function of the number and duration of events included in the videos (central tendency and distribution)

Number of Events	Event Duration	N	Q1	Median	Q3	Skew	Kurtosis
**One**							
	3	68	2.86	3.71	4.47	-0.25	-0.74
	6	68	5.05	6.45	7.72	-0.23	-0.32
	9	71	6.12	8.62	10.05	-0.07	-0.62
	12	72	6.53	9.87	12.68	0.02	-1.01
**Two**							
	3	70	2.61	3.25	3.66	0.25	0.14
	6	72	4.25	5.27	6.23	0.16	0.13
	9	72	4.65	6.74	8.95	0.12	-0.77
	12	71	5.99	8.07	11.52	-0.12	-1.10
**Three**							
	3	71	2.46	3.38	4.37	-0.08	-0.92
	6	71	3.88	5.35	6.64	-0.07	-0.63
	9	72	4.67	6.23	8.77	0.35	-0.55
	12	71	5.46	8.12	11.00	0.02	-1.07

Table S2

Event temporal ratio as a function of the number and duration of events included in the videos (central tendency and distribution).

Number of Events	Event Duration	N	Q1	Median	Q3	Skew	Kurtosis
**One**							
	3	68	0.95	1.24	1.49	-0.25	-0.74
	6	68	0.84	1.07	1.29	-0.23	-0.32
	9	71	0.68	0.96	1.12	-0.07	-0.62
	12	72	0.54	0.82	1.06	0.02	-1.01
**Two**							
	3	70	0.87	1.08	1.22	0.25	0.14
	6	72	0.71	0.88	1.04	0.16	0.13
	9	72	0.52	0.75	0.99	0.12	-0.77
	12	71	0.50	0.67	0.96	-0.12	-1.10
**Three**							
	3	71	0.82	1.13	1.46	-0.08	-0.92
	6	71	0.65	0.89	1.11	-0.07	-0.63
	9	72	0.52	0.69	0.97	0.35	-0.55
	12	71	0.45	0.68	0.92	0.02	-1.07

Main effects analyses

Following our pre-registered plan, we initially assessed the main effects of the number and duration of events using models that did not include the interaction terms as a predictor. Subsequently, we evaluated the interactions in separate models. Due to the observed interaction effect in the model predicting event remembering duration and to maintain conciseness, we have only included the results of the models with interactions in the main text. For the sake of completeness, the complete analysis of main effects is reported here.

We examined the main effects of event number and duration on event remembering duration and event temporal ratio by means of two growth curve analyses (Mirman, 2014; Winter & Wieling, 2016) with event number treated as a 3-level factor and event duration transformed in first and second order orthogonal polynomials (i.e., linear and quadratic terms) as predictors.

The first growth curve predicted event remembering duration and included the main effects of event number and duration as predictors as well as two correlated random effects: a random intercept for participants and a random slope for the linear term (see Figure S1). The model explained 76% of the variance in event remembering duration (Conditional R^2 = 0.76) and its fixed part 39% (Marginal R^2 = 0.39). As expected, event remembering duration increased with event duration (i.e., the longer an event, the longer the event remembering duration), but not proportionally (linear term: b =3.87, 95% *CI* [3.31, 4.43], t = 13.57, $p < 0.001$; quadratic term: b = -0.32, 95% *CI* [-0.49, -0.15], t = -3.63, $p < 0.001$). In addition, event remembering duration was higher when events were presented in isolation than when they were included in videos that contained several events (one event vs. two events: b = 0.95, 95% *CI*[0.69, 1.21], t = 8.89, $p < 0.001$; one event vs. three event: b = 1.10, 95% *CI* [0.84, 1.35], t = 10.27, $p < 0.001$); event remembering duration did not differ significantly depending on

whether two or three events had to be mentally replayed (two vs. three events: $b = 0.15$, 95% CI [-0.11, 0.40], $t = 1.40$, $p = 0.49$).

Figure S1

Main effects of event number and duration on event remembering duration

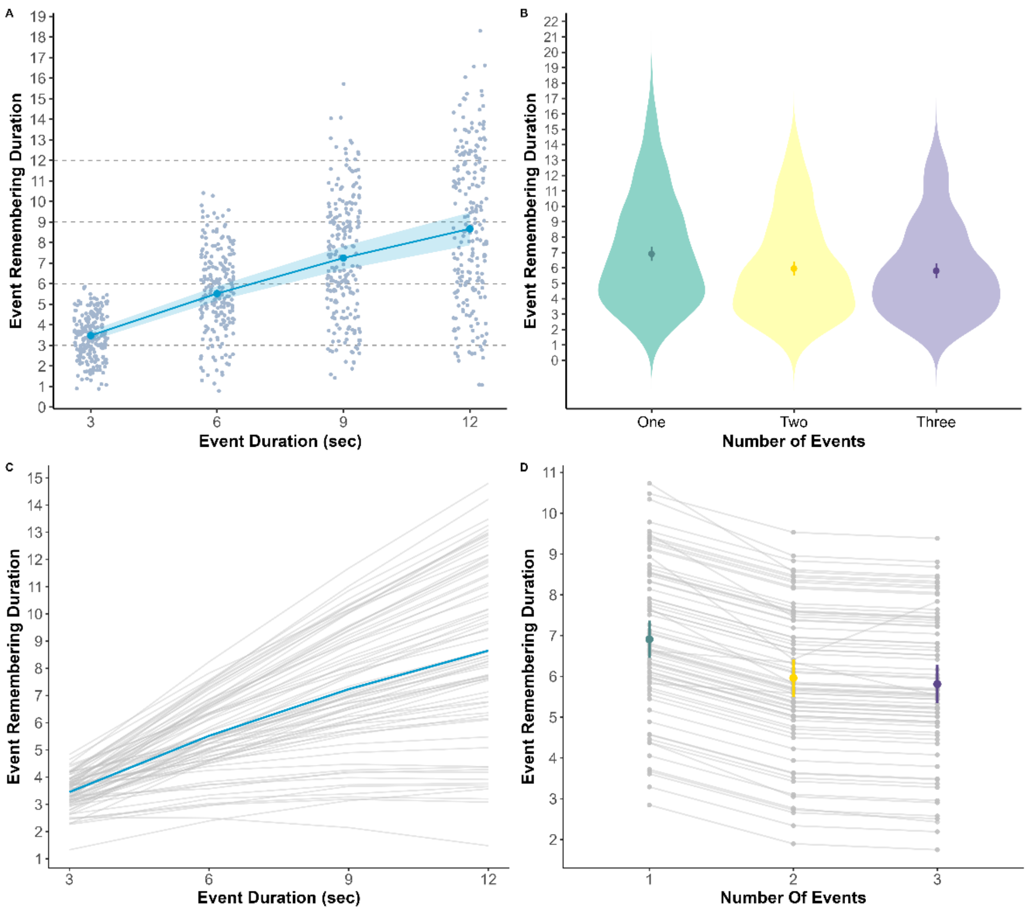

Note. **A.** Evolution of event remembering duration with event duration. The gray dots represent the observed values. Each point corresponds to one observed value. The blue-green dots and the ribbon surrounding them represent the estimated marginal means and their 95% CIs for each event duration (averaged over the different event numbers). **B.** Event remembering duration as a function of event number. The violins depict the distribution of observed values. Dots and vertical bars surrounding them correspond to the estimated marginal means and their 95% CIs for each event number (averaged over the event durations). **C.** *Increase of event remembering duration with event duration*. Each grey line corresponds to the estimated slope of a single participant. The blue line represents the global trend according to the model estimates. **D.** *Estimated event remembering duration across event numbers*. The colored dots depict the estimated means and their 95 % *CI*s for each number of events. The grey dots and lines depict estimated remembering durations across the different number of events for each participant.

The second growth curve had the same predictors but event temporal ratio as outcome (see Figure S2). The model explained 75% of the variance in event temporal ratio (Conditional $R^2 = 0.75$) and its fixed part 22% (Marginal $R^2 = 0.22$). This analysis revealed a significant (non-linear) decrease of event temporal ratio with event duration (linear term: $b = -0.30$, 95% *CI* [-0.35, -0.25], $t = 11.02$, $p < 0.001$; quadratic term: $b = 0.06$, 95% *CI* [0.04, 0.09], $t = 4.81$, $p < 0.001$). Event temporal ratio was above 1 for short events but smaller for longer events (Figure S2). Concerning the effect of event number, event temporal ratio was higher for events presented alone than in a sequence (one event vs. two events: $b = 0.15$, 95% *CI* [0.11, 0.18], $t = 9.30$, $p < 0.001$; one event vs. three event: $b = 0.16$, 95% *CI* [0.13, 0.2], $t = 10.48$, $p < 0.001$) but did not differ significantly depending on whether two or three events had to be mentally replayed ($b = 0.02$, 95% *CI* [-0.02, 0.06], $t = 1.20$, $p = 0.69$).

Figure S2

Main effects of event number and duration on event temporal ratio

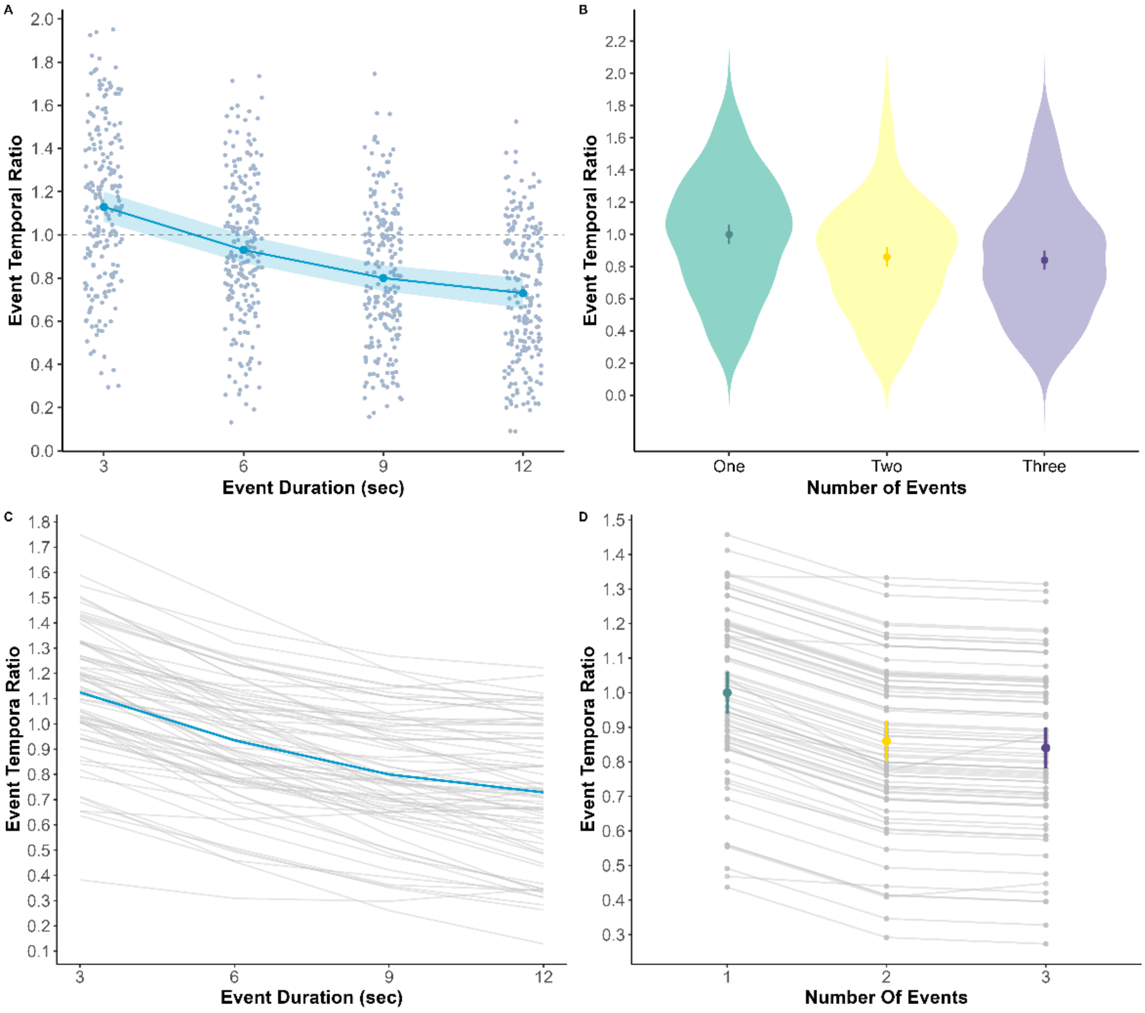

Note. **A.** Evolution of event temporal ratio with event duration. The gray dots represent the observed values. Each point corresponds to one observed value. The blue-green dots and the ribbon surrounding them represent the estimated marginal means and their 95% CIs for each event duration (averaged over the different event numbers). **B.** Event temporal ratio as a function of event number. The violins depict the distribution of observed values. Dots and vertical bars surrounding them correspond to the estimated marginal means and their 95% CIs for each event number (averaged over the event durations). **C.** *Decrease of event temporal ratio with event duration*. Each grey line corresponds to the estimated slope of a single participant. The blue line represents the global trend according to the model estimates. **D.** *Estimated event temporal ratio across event numbers*. The colored dots depict the estimated means and their 95 % *CI*s for each number of events. The grey dots and lines depict estimated temporal ratios across the different number of events for each participant.

Supplementary analyses

To further characterize the effects of the number and duration of events on the temporal compression of memories, we conducted some additional (non-registered) analyses. These are summarized in the Main Text and reported in detail below.

Effect of event number on memory replay for the entire video

We evaluated whether participants took significantly more time to remember videos composed of a larger number of events (i.e., whether mental replay times for the entire video clips increased with the number of events they contained). This was indeed the case: on average, mental replay durations were greater when videos included more events. Importantly, this was observed in most participants and for all event durations (see Figure S3).

Figure S3

Differences in remembering duration for the entire video clips depending on the number of events they contained

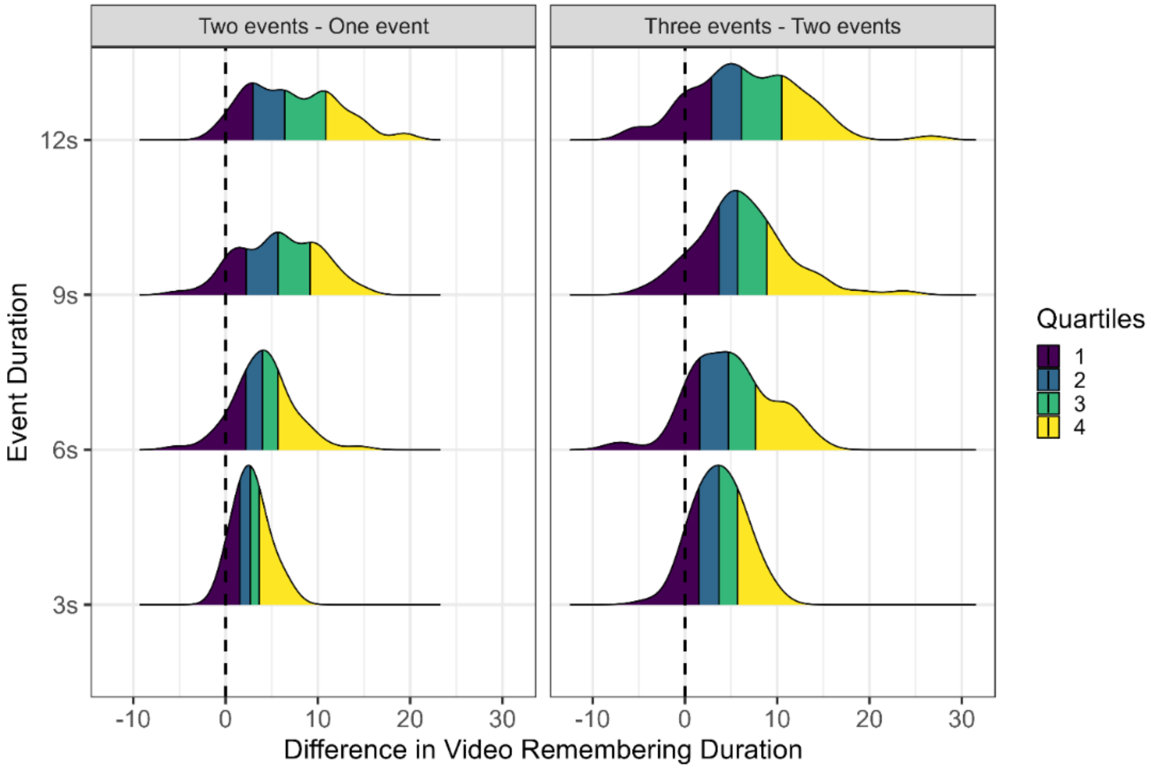

Note. Differences in remembering duration between videos containing two events and those including a single event were positive (i.e., remembering two-events videos took more time than remembering single-event videos) in more than 75% of participants and for each event duration (left panel). Differences in remembering duration between videos containing three events and those including two events were positive (i.e., remembering three-events videos took more time than remembering two-event videos) in more than 75% of participants and for each event duration (right panel).

We then assessed the statistical significance of this increase of video remembering duration with event number. We fitted a model with the remembering duration for the whole videos (in seconds) as outcome and two fixed predictors: the number of events (treated as a 3-level factor) and event duration transformed in first and second order orthogonal polynomials. This model included three uncorrelated random effects: one intercept for participants, one random slope for the effect of event number and a random slope for the effect of the linear term

(see Figure S4). The model explained 82% of the variance in remembering duration (Conditional R^2 = 0.82) and its fixed part 67% (Marginal R^2 = 0.67). The differences in remembering duration between single-event and two-event videos, and between two-event and three-event videos, were both statistically significant (*b* = -4.86, 95% *CI* [-5.63, -4.08], *t* = -14.99, *p* <0.001; *b* = -5.11, 95% *CI* [-6.43, -3.79], *t* = -9.29, *p* <0.001).

Figure S4

Effect of the number of events on remembering duration for the whole videos: intercept and slope of each participant

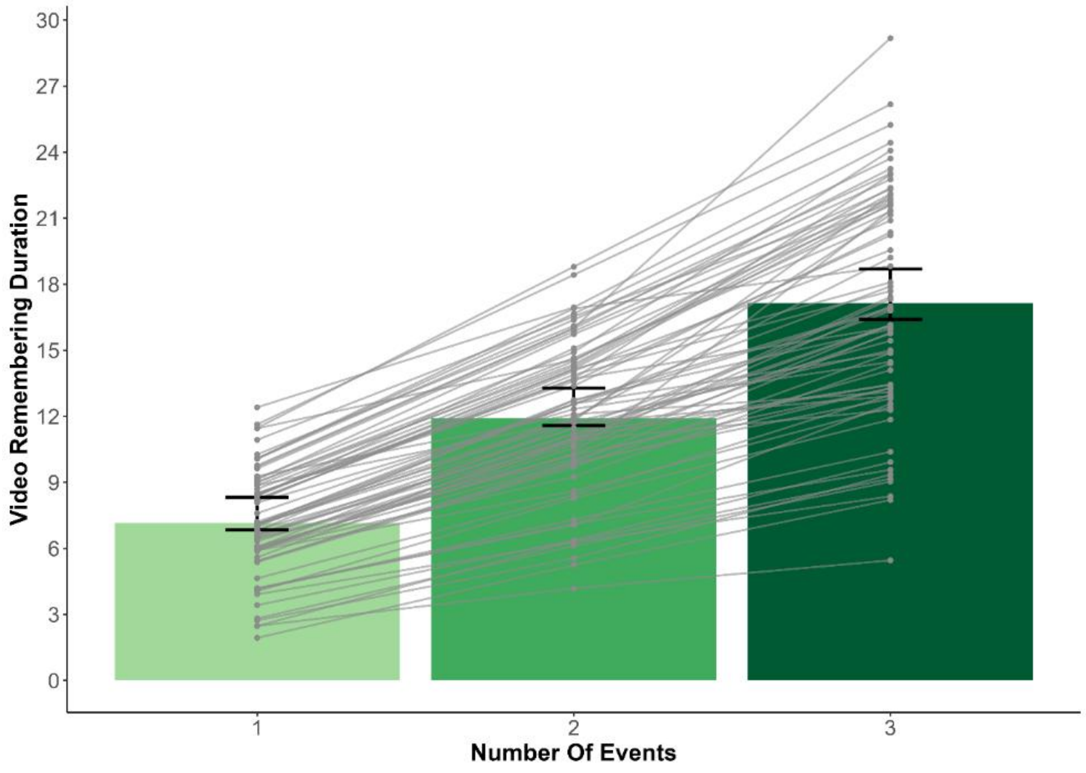

Note. Green bars and corresponding error bars represent estimated means and their 95% *CI*s for each event number. The grey dots and lines depict estimated remembering durations across the different number of events for each participant.

Effect of event number on temporal ratio when the total video duration is kept constant

While the present study was not specifically designed for this purpose, we analyzed a subset of data to examine whether, for a given video duration, the temporal ratio varied depending on the number of events the video contained. We first selected video durations for which we had stimuli with either one or several (i.e., two or three) events. This selection left us with 567 observations (see Figure S5). We then fitted a robust linear mixed-effect model predicting video temporal ratio (i.e., video remembering duration divided by the video duration) by event number, video duration and their interaction. Event number and duration were both treated as continuous variables and mean-centered to avoid collinearity issues. Video duration was divided by ten to reduce the differences between predictor scales. The model included a random intercept for participants and a random slope for the effect of the number of events.

First, the video temporal ratio significantly decreased (i.e., temporal compression increased) with video duration (b = -0.28, 95% *CI* [-0.32, -0.24], t = -12.31, p < 0.001). In addition, there was a main effect of event number such that the higher the number of events, the higher the temporal ratio (b = 0.08, 95% *CI* [0.06, 0.11], t = 6.44, p < 0.001). Finally, there was also a significant interaction (b =0.07, 95% *CI* [0.01, 0.13], t = 2.18, p = 0.029), revealing that the effect of event number on the temporal ratio was stronger for longer videos.

Figure S5

Effect of the number of events on video temporal ratio (by video duration)

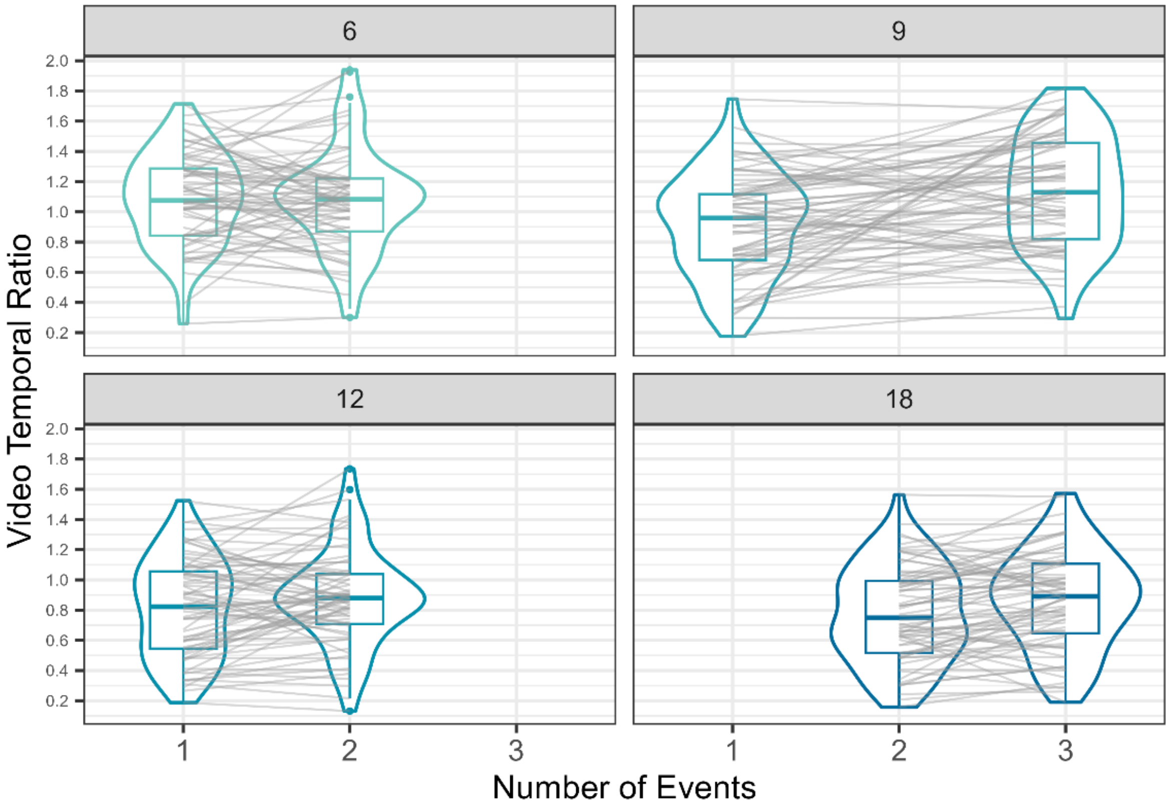

*Note***.** Boxes and violins depict the distribution of observed values (across participants). Within each video duration (6, 9, 12 or 18 s), each gray lines represent the observed value for a single participant. For a given video duration, the video temporal ratio tended to be higher (i.e., temporal compression was lower) when the video contained a greater number of events.

Model comparisons

We further ensured that the observed effects of event number and duration were not merely driven by their correlation with total stimulus duration, using a model comparison approach. We compared the goodness-of-fit of models predicting event remembering duration, event temporal ratio, total remembering duration, and total temporal ratio. For each outcome, we contrasted two models: a model including a first and a second order polynomial transformation of event duration, event number (treated as a three-level factor) and their interaction as predictors, and a model including a first and a second order polynomial transformation of total stimulus duration as predictors.

We compared the goodness of fit of the two models on seven indices (Figure S6). First, the marginal and conditional Nakagawa's R-squared (R2s). Marginal R2 represents the part of the dependent variable variance explained by fixed effects alone, while conditional R2 represents the variance explained by the entire model (fixed and random effects; Johnson, 2014; Nakagawa et al., 2017; Nakagawa & Schielzeth, 2013). The part of variance explained by the grouping structure of our data was quantified with adjusted intra-class correlation (ICC; Hox, 2010; Rabe-Hesketh & Skrondal, 2012; Raudenbush & Bryk, 2002). The higher these three indices, the better the model fit. The accuracy and precision of models' predicted values were assessed using the Root Mean Square Error (RMSE; Chai & Draxler, 2014; Kenney & Keeping, 1962) and the residual standard deviation (sigma; $\boldsymbol{\sigma}$; Gelman et al., 2020). Finally, we assessed the balance between the amount of explained variance and the number of predictors in the model through Akaike Information Criterion (AIC; Sakamoto et al., 1984) and Bayesian Information Criterion (BIC; Schwarz, 1978). The lower these four indices, the better the model fit.

The model with event number and duration as predictors outperformed the model with total video duration as single predictor on all indices (see Figure S6).

Figure S6

Spider chart of model performance indices

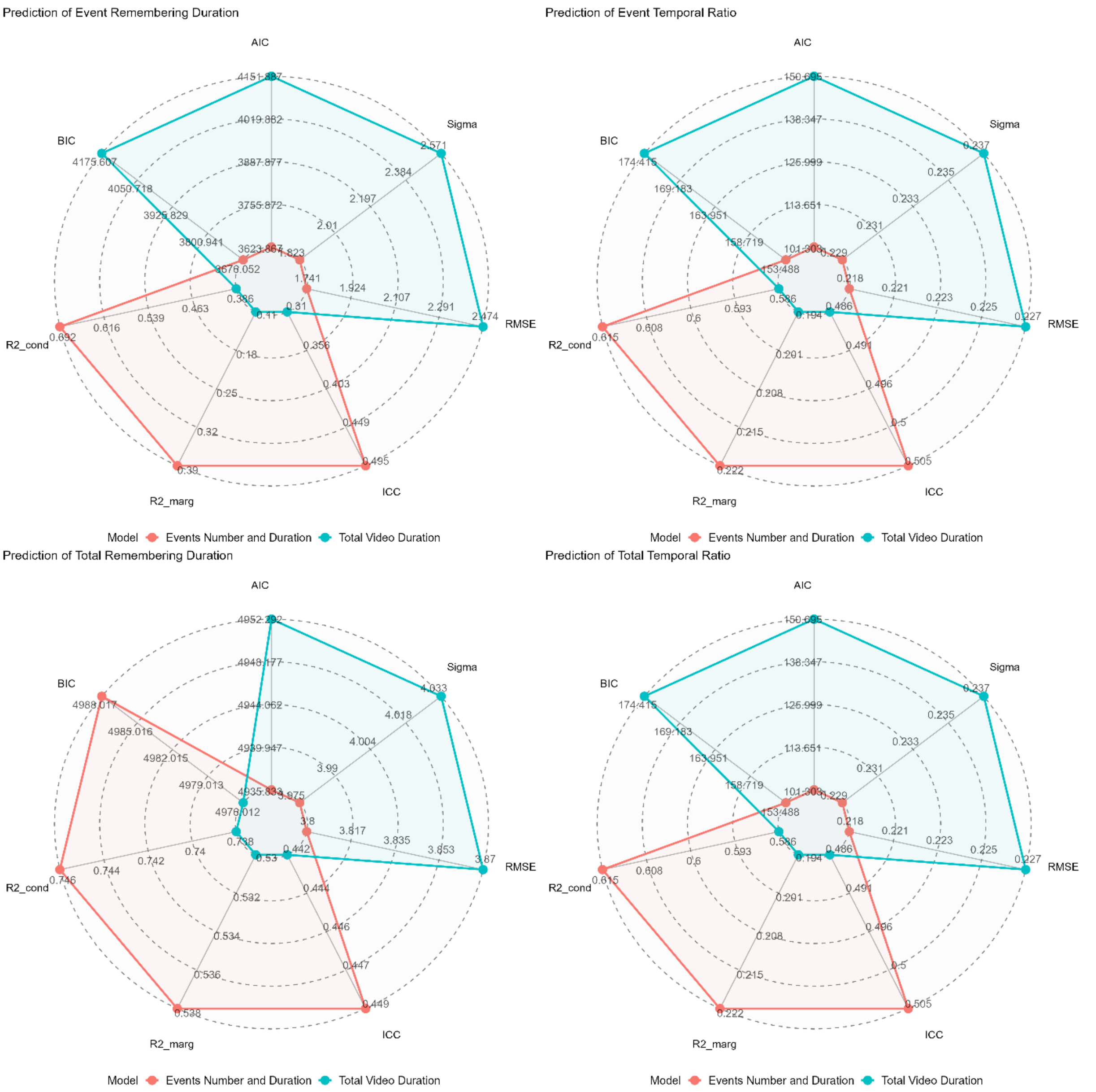

Note. Indices assess how well models predicting event remembering duration (top left), event temporal ratio (top right), total remembering duration (bottom left) and total temporal ratio (bottom right) by either event number and duration (red polygon) or by total event duration (blue-green polygon) fitted the data.

Supplementary information about exploratory analyses on the role of visual imagery

Distribution and reliability assessment of VVIQ scores

A quick look at items' response frequency (Figure S7A) showed that the 1 and 2 responses were almost never used, suggesting that most participants were able to produce some form of visual images of the described scenes. All but one of the participants responded to all the items. Participants' total VVIQ scores ranged between 43 and 77 (Figure S7B).

Figure S7

VVIQ score: distribution and reliability

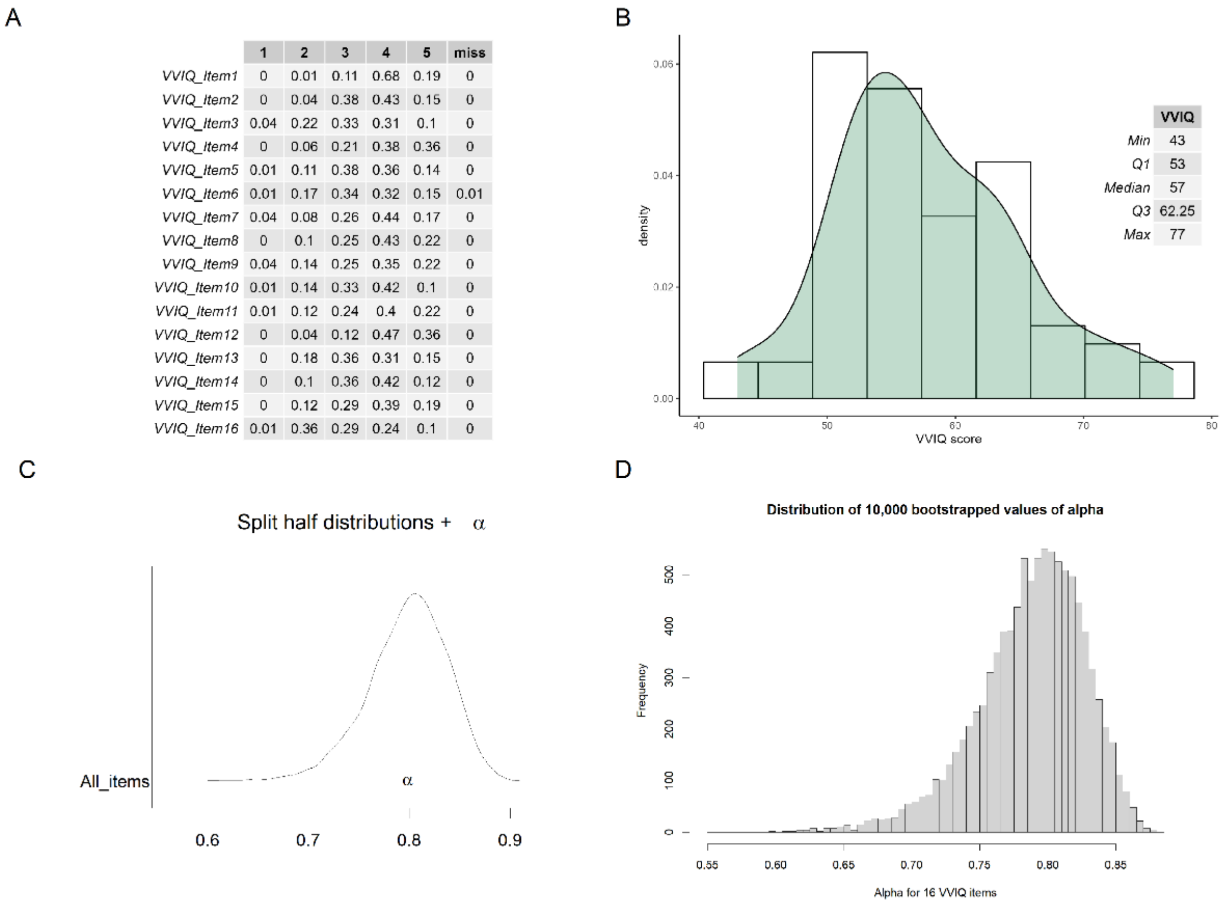

	1	2	3	4	5	miss
VVIQ_Item1	0	0.01	0.11	0.68	0.19	0
VVIQ_Item2	0	0.04	0.38	0.43	0.15	0
VVIQ_Item3	0.04	0.22	0.33	0.31	0.1	0
VVIQ_Item4	0	0.06	0.21	0.38	0.36	0
VVIQ_Item5	0.01	0.11	0.38	0.36	0.14	0
VVIQ_Item6	0.01	0.17	0.34	0.32	0.15	0.01
VVIQ_Item7	0.04	0.08	0.26	0.44	0.17	0
VVIQ_Item8	0	0.1	0.25	0.43	0.22	0
VVIQ_Item9	0.04	0.14	0.25	0.35	0.22	0
VVIQ_Item10	0.01	0.14	0.33	0.42	0.1	0
VVIQ_Item11	0.01	0.12	0.24	0.4	0.22	0
VVIQ_Item12	0	0.04	0.12	0.47	0.36	0
VVIQ_Item13	0	0.18	0.36	0.31	0.15	0
VVIQ_Item14	0	0.1	0.36	0.42	0.12	0
VVIQ_Item15	0	0.12	0.29	0.39	0.19	0
VVIQ_Item16	0.01	0.36	0.29	0.24	0.1	0

Note. **A.** Frequency of each possible response (from 1 to 5) and nonresponse ("misses") for each of the 16 VVIQ items. **B.** Density plot of VVIQ scores across participants and related descriptive statistics. **C.** Distribution of the split-half reliabilities obtained across all possible 8 item splits (i.e., 6,435) and observed Cronbach's alpha. **D.** Distribution of the coefficient α obtained across resampling.

In our sample, the observed Cronbach's alpha (α; Cronbach, 1951) was 0.79 (see Fig S7C). The 95% confidence interval computed using normal theory (Feldt et al., 1987) was from 0.72 to 0.86[1]. The empirical bootstrapped 95% confidence interval (Revelle & Condon, 2018) was quite similar (i.e., from 0.69 to 0.85; see Figure S7D). Although there is no real consensus on which value constitutes an appropriate α, various authors agree that, for scales used as research tools, α coefficient should lie between 0.6 and 0.9 (values below 0.6 reflecting a clear lack of internal consistency, and values above 0.9 suggesting unnecessary redundancies within the scale; Streiner, 2003; Taber, 2018).

The average inter-item correlation was 0.2, which, according to Clark and Watson, (1995) is adequate for scales assessing broad psychological constructs (i.e., it lies between 0.15 and 0.20). The average Guttman's lambda 6 (λ6; the amount of an item's variance which is predictable by all of the other; Guttman, 1945) was 0.84.

Relations between VVIQ scores and memory measures

We expected to observe a positive association between participants' vividness of visual imagery and their propensity to remember events with a duration close to their actual duration (i.e., to find a positive association between VVIQ scores and event remembering duration, event temporal ratio, and the stimuli total remembering duration). Moreover, we expected that individuals with higher vividness of visual imagery would be less subject to the deleterious effects of event number and duration on event remembering duration and event temporal ratio.

---

[1] The relatively wide range of this interval can be explained by our sample size, which was small to perform this kind of inference in a precise way. As an example, according to equation 8 from Bonett (2002), under conditions similar to those in this study, a sample size of about 150 participants would be needed to estimate α with a precision (upper bound of exact confidence interval - lower bound of exact confidence interval) of 0.1 (Arifin, 2024).

We investigated these possibilities with three robust linear mixed-effect models (Koller, 2016). The first model predicted event remembering duration by event duration, event number, VVIQ scores, and their interactions. The two other models were fitted with the same predictors but had respectively event temporal ratio and video total remembering duration as outcome. In all the models, event duration was transformed in first and second order orthogonal polynomials (i.e., linear and quadratic terms), event number was treated as a 3-level factor, and VVIQ scores were transformed into *z*-scores. The three models included the same random effects: a random intercept for participants and a random slope (at the participant level) for the linear term.

The main results of the first two models are described in the main text. We observed that the increase in event remembering duration with event duration was stronger for participants with higher VVIQ scores. Coherently, the decrease of event temporal ratio with event duration was weaker for participants with higher VVIQ scores. More details about observed and estimated means are reported in Figure S8 and Table S3**.**

Taken together, these results suggest that individuals with higher visual imagery are able to more accurately remember the unfolding of continuous events. This tendency was further confirmed by the analysis of stimuli total remembering duration (Figure S8C and right part of Table S3). First, the increase of stimuli total remembering duration as a function of the number of events was stronger in individuals with high VVIQ scores (interaction between linear term and VVIQ scores: b = 1.13, 95% *CI* [0.09, 2.16]). In addition, this interaction effect (event number and VVIQ scores) was more pronounced for stimuli composed of longer events (three-way interaction: b = 1.48, 95% *CI* [0.48, 2.47]).

Figure S8

Effects of event number and duration as a function of VVIQ score

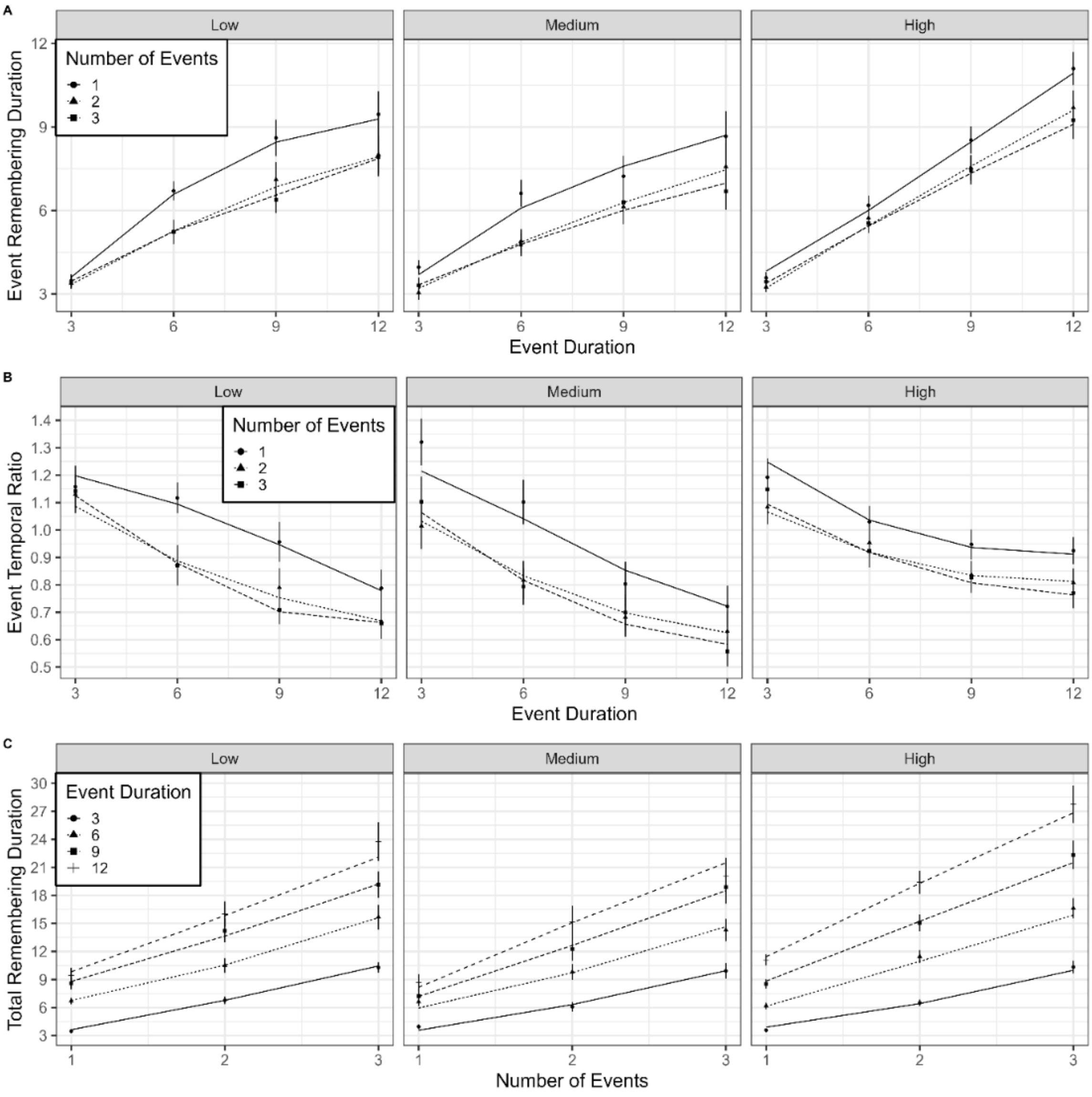

Note. Prediction of event remembering duration (**A**), event temporal ratio (**B**) and stimuli total remembering duration (**C**) by event duration, event number, VVIQ score and their interactions. Observed data (symbols, vertical lines indicate +- *SE*) and model fits (lines). The VVIQ score predictor was discretized (i.e., scores were classified into three categories: "low", "medium" and "high") for illustration purpose (Mirman, 2014). On the figure, the "low" facets depict the observed and fitted values for participants with a VVIQ score between 43 and 54 (N participants = 26, N trials = 304), the “medium” facets relate to participants with a score between 55 and 60 (N participants = 22, N trials = 259), the “high” facets refer to participants with a score between 61 and 77 (N participants = 24, N trials = 286).

Table S3

Estimated means as a function of event duration, event number and VVIQ score

Event Duration	Event Number	VVIQ	ERD	95% CI ERD	ETR	95% CI ETR	TRD	95% CI TRD
3	One	-1 SD	3.70	[3.21, 4.18]	1.23	[1.12, 1.34]	3.69	[2.62, 4.76]
6	One	-1 SD	6.52	[5.95, 7.08]	1.09	[1.00, 1.18]	6.58	[5.42, 7.74]
9	One	-1 SD	8.29	[7.45, 9.12]	0.93	[0.84, 1.03]	8.45	[6.81, 10.09]
12	One	-1 SD	9.01	[7.84, 10.17]	0.75	[0.64, 0.86]	9.32	[7.05, 11.58]
3	Two	-1 SD	3.38	[2.89, 3.87]	1.09	[0.99, 1.20]	6.74	[5.66, 7.82]
6	Two	-1 SD	5.19	[4.63, 5.75]	0.88	[0.79, 0.98]	10.35	[9.20, 11.51]
9	Two	-1 SD	6.68	[5.84, 7.51]	0.74	[0.64, 0.83]	13.30	[11.66, 14.94]
12	Two	-1 SD	7.83	[6.67, 9.00]	0.66	[0.54, 0.77]	15.57	[13.30, 17.84]
3	Three	-1 SD	3.47	[2.99, 3.96]	1.13	[1.02, 1.23]	10.41	[9.34, 11.48]
6	Three	-1 SD	5.08	[4.51, 5.64]	0.86	[0.76, 0.95]	15.28	[14.12, 16.44]
9	Three	-1 SD	6.38	[5.54, 7.21]	0.69	[0.59, 0.78]	18.86	[17.22, 20.50]
12	Three	-1 SD	7.38	[6.21, 8.54]	0.62	[0.51, 0.73]	21.16	[18.89, 23.44]
3	One	+1 SD	3.78	[3.29, 4.26]	1.23	[1.12, 1.34]	3.76	[2.69, 4.83]
6	One	+1 SD	5.93	[5.36, 6.50]	1.02	[0.93, 1.12]	5.94	[4.76, 7.11]
9	One	+1 SD	8.13	[7.29, 8.97]	0.90	[0.80, 1.00]	8.16	[6.50, 9.81]
12	One	+1 SD	10.36	[9.19, 11.54]	0.86	[0.75, 0.98]	10.42	[8.13, 12.70]
3	Two	+1 SD	3.15	[2.67, 3.63]	1.04	[0.93, 1.14]	6.27	[5.22, 7.33]
6	Two	+1 SD	5.24	[4.67, 5.81]	0.88	[0.79, 0.98]	10.50	[9.33, 11.66]

Event Duration	Event Number	VVIQ	ERD	95% CI ERD	ETR	95% CI ETR	TRD	95% CI TRD
9	Two	+1 SD	7.21	[6.37, 8.05]	0.79	[0.70, 0.89]	14.46	[12.80, 16.11]
12	Two	+1 SD	9.05	[7.87, 10.22]	0.76	[0.65, 0.88]	18.16	[15.87, 20.45]
3	Three	+1 SD	3.32	[2.84, 3.80]	1.06	[0.96, 1.17]	9.85	[8.79, 10.91]
6	Three	+1 SD	5.21	[4.65, 5.78]	0.88	[0.79, 0.97]	15.45	[14.28, 16.62]
9	Three	+1 SD	6.95	[6.11, 7.79]	0.76	[0.67, 0.86]	20.70	[19.04, 22.35]
12	Three	+1 SD	8.54	[7.36, 9.72]	0.72	[0.60, 0.83]	25.59	[23.30, 27.88]

Note. Estimates means (and 95% CIs) for event remembering duration (ERD), event temporal ratio (ETR) and stimuli total remembering duration (TRD) as a function of event duration, event number, and VVIQ *Z*-score (-1 standard deviation vs. +1 standard deviation).

Interestingly, the stronger increase of stimuli total remembering duration with the number of events in individuals with higher VVIQ scores was not observed for stimuli composed of short events (i.e., 3 and 6 s; see Figure S8 and Table S3). Furthermore, for stimuli composed of 3-s events (which tended, on average, to be remembered with a duration slightly higher than their actual one), the effect of event number on total remembering duration was negatively correlated with VVIQ scores (see Figure S9). This pattern of results suggests that, in addition to enabling a more complete mental replay of longer events, higher visual imagery abilities could enable a faster retrieval/reconstruction of shorter events.

The correlation matrix displayed in Figure S9 also reveals that individual differences in the magnitude of the increase of stimuli total remembering duration with the number of events were strongly correlated across event durations, except for the stimuli composed of 3-s events. This could suggest that the remembering of sequence of events involves different cognitive mechanisms depending on whether they are composed of sub-events whose duration exceeds

3-s. This pattern echoes research on temporal cognition suggesting that the “subjective present” lasts about 3 s, which would correspond to the elementary units of the flow of consciousness (Fairhall et al., 2014; Monfort et al., 2020; Montemayor & Wittmann, 2014; Pöppel, 1997). It is only beyond this duration that WM would become involved to enable the maintenance of several of these units of “now” in an active state to form more complex event representations (i.e., event models; Richmond & Zacks, 2017).

Figure S9

Exploratory correlation matrix.

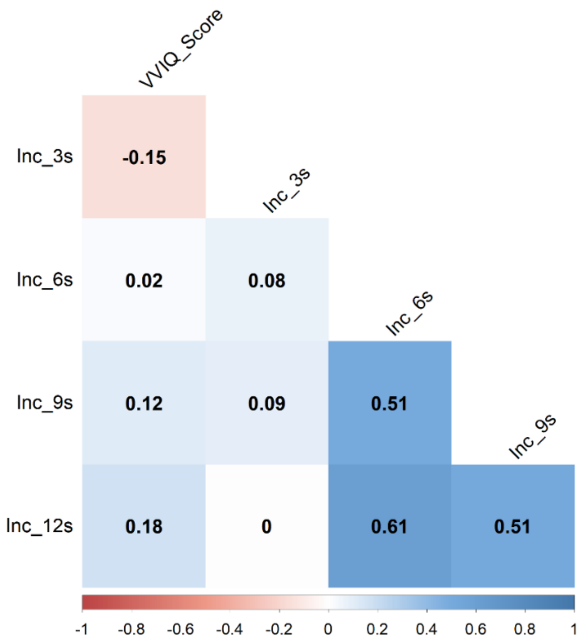

Note. Spearman correlation between VVIQ scores and the increase in stimuli total remembering duration (TRD) with the number of events (for the four possible event durations). Data were averaged by event duration for each participant. The magnitude of the effect of event number on TRD was quantified as follow: (TRD 2events – TRD 1event) + (TRD 3events – TRD 1event) + (TRD 3events – TRD 2event). Colors refer to the direction and the size of the correlation (*rho*): blue refers to positive associations whereas red refers to negative associations; the darker the color, the stronger the correlation.

R packages

The R project was built with renv (version 0.17.3; Ushey & Wickham, 2023). Analyses scripts were written using rmarkdown (version 2.17; Xie et al., 2020). Bibliographical information relating to the R packages was obtained from packages report (version 0.5.7; Makowski et al., 2023) and knitr (version 1.40; Xie, 2015).

Tables were made with the packages dplyr (version 1.1.3; Wickham et al., 2023), flextable (version 0.9.3) and rempsyc (version 0.1.1; Thériault, 2023). Figures were made with the packages ggplot2 (version 3.4.4; Wickham, 2016), ggridges (version 0.5.6; Wilke, 2024), gridExtra (version 2.3; Auguie, 2017), ggvanced (Zvonimir, 2023), ggpubr (version 0.6.0; Kassambara, 2023) and corrplot (version 0.92; Wei & Simko, 2021). Some of the descriptive statistics and reliability indices were computed with the psych package (version 2.3.9; Revelle, 2023).

The power analysis for this study was conducted with the help of the R package simr (version 1.0.7; Green & MacLeod, 2016; Kumle et al., 2021). Application conditions of classical linear mixed-effects models were checked with the performance package (version 0.10.5; Lüdecke et al., 2023). The robust estimation procedure (the DAStau procedure) was implemented using the robustlmm package (version 3.0.4; Koller, 2016).

We relied on functions from the parameters (version 0.21.2; Lüdecke et al., 2020) to compute confidence intervals (*CIs*) and *p*-values (for fixed effects) from standard errors (*SEs*) and *t*-statistics. Estimated means, associated pointwise standard errors and Wald's 95% CIs were computed with the help of the package effects (version 4.2.2;J. Fox & Weisberg, 2019) and emmeans (version 1.8.8; Lenth, 2016).

References

Arifin, W. N. (2024). *Sample size calculator* [Computer software]. Retrieved from http://wnarifin.github.io

Auguie, B. (2017). *gridExtra : Miscellaneous functions for « Grid » graphics* [Computer software]. https://CRAN.R-project.org/package=gridExtra

Bonett, D. (2002). Sample size requirements for testing and estimating coefficient alpha. *Journal of Educational and Behavioral Statistics - J EDUC BEHAV STAT*, *27*, 335-340. https://doi.org/10.3102/10769986027004335

Chai, T., & Draxler, R. R. (2014). Root mean square error (RMSE) or mean absolute error (MAE)? – Arguments against avoiding RMSE in the literature. *Geoscientific Model Development, 7*(3), 1247-1250. https://doi.org/10.5194/gmd-7-1247-2014

Clark, L. A., & Watson, D. (1995). Constructing validity : Basic issues in objective scale development. *Psychological Assessment, 7*(3), 309-319.

Cronbach, L. J. (1951). Coefficient alpha and the internal structure of tests. *Psychometrika, 16*(3), 297-334. https://doi.org/10.1007/BF02310555

Fairhall, S. L., Albi, A., & Melcher, D. (2014). Temporal Integration Windows for Naturalistic Visual Sequences. *PLoS ONE, 9*(7), e102248. https://doi.org/10.1371/journal.pone.0102248

Feldt, L. S., Woodruff, D. J., & Salih, F. A. (1987). Statistical inference for coefficient alpha. *Applied Psychological Measurement*, *11*(1), 93-103.

Fox, J., & Weisberg, S. (2019). *An R Companion to Applied Regression* (3rd éd.). Sage. https://socialsciences.mcmaster.ca/jfox/Books/Companion/index.html

Gelman, A., Hill, J., & Vehtari, A. (2020). *Regression and other stories*. Cambridge University Press. https://doi.org/10.1017/9781139161879

Green, P., & MacLeod, C. J. (2016). simr : An R package for power analysis of generalised linear mixed models by simulation. *Methods in Ecology and Evolution*, *7*(4), 493-498. https://doi.org/10.1111/2041-210X.12504

Guttman, L. (1945). A basis for analyzing test-retest reliability. *Psychometrika, 10*, 255-282. https://doi.org/10.1007/BF02288892

Hox, J. J. (2010). *Multilevel analysis : Techniques and applications* (2. ed). Routledge, Taylor & Francis.

Johnson, P. C. D. (2014). Extension of Nakagawa & Schielzeth's R^2_{GLMM} to random slopes models. *Methods in Ecology and Evolution*, *5*(9), 944-946. https://doi.org/10.1111/2041-210X.12225

Kassambara, A. (2023). *ggpubr : Ggplot2 based publication ready plots* [Computer software]. https://rpkgs.datanovia.com/ggpubr/

Kenney, J. F., & Keeping, E. S. (1962). Root Mean Square. In *Mathematics of Statistics* (3rd ed, p. 59-60). Princeton.

Koller, M. (2016). robustlmm : An R Package for Robust Estimation of Linear Mixed-Effects Models. *Journal of Statistical Software*, *75*(6), 1-24. https://doi.org/10.18637/jss.v075.i06

Kumle, L., Võ, M. L.-H., & Draschkow, D. (2021). Estimating power in (generalized) linear mixed models : An open introduction and tutorial in R. *Behavior Research Methods*, *53*(6), 2528-2543. https://doi.org/10.3758/s13428-021-01546-0

Lenth, R. V. (2016). Least-Squares Means : The *R* Package **lsmeans**. *Journal of Statistical Software, 69*(1). https://doi.org/10.18637/jss.v069.i01

Lüdecke, D., Ben-Shachar, M. S., Patil, I., & Makowski, D. (2020). Extracting, computing and exploring the parameters of statistical models using R. *Journal of Open Source Software*, *5*(53), 2445. https://doi.org/10.21105/joss.02445

Lüdecke, D., Makowski, D., Ben-Shachar, M. S., Patil, I., Waggoner, P., & Wiernik, B. M. (2023). *performance : Assessment of regression models performance* [Computer software]. https://easystats.github.io/performance/

Makowski, D., Lüdecke, D., Patil, I., Thériault, R., Ben-Shachar, M. S., & Wiernik, B. M. (2023). *report : Automated reporting of results and statistical models* [Computer software]. https://easystats.github.io/report/

Mirman, D. (2014). *Growth curve analysis and visualization using R* (1st éd.). Chapman and Hall/CRC. https://doi.org/10.1201/9781315373218

Monfort, M., Vondrick, C., Oliva, A., Andonian, A., Zhou, B., Ramakrishnan, K., Bargal, S. A., Yan, T., Brown, L., Fan, Q., & Gutfreund, D. (2020). Moments in Time Dataset : One Million Videos for Event Understanding. *IEEE Transactions on Pattern Analysis and Machine Intelligence*, *42*(2), 502-508. https://doi.org/10.1109/TPAMI.2019.2901464

Montemayor, C., & Wittmann, M. (2014). The Varieties of Presence : Hierarchical Levels of Temporal Integration. *Timing & Time Perception*, *2*(3), 325-338. https://doi.org/10.1163/22134468-00002030

Nakagawa, S., Johnson, P. C. D., & Schielzeth, H. (2017). The coefficient of determination R2 and intra-class correlation coefficient from generalized linear mixed-effects models revisited and expanded. *The Royal Society Publishing*, *14*. http://dx.doi.org/10.1098/rsif.2017.0213

Nakagawa, S., & Schielzeth, H. (2013). A general and simple method for obtaining R^2 from generalized linear mixed-effects models. *Methods in Ecology and Evolution*, *4*(2), 133-142. https://doi.org/10.1111/j.2041-210x.2012.00261.x

Pöppel, E. (1997). A hierarchical model of temporal perception. *Trends in Cognitive Sciences*, *1*(2), 56-61. https://doi.org/10.1016/S1364-6613(97)01008-5

Rabe-Hesketh, S., & Skrondal, A. (2012). *Multilevel and longitudinal modeling using Stata* (3rd ed). Stata Press Publication.

Raudenbush, StephenW., & Bryk, A. S. (2002). *Hierarchical Linear Models Applications and Data Analysis Methods* (2e éd.). Sage Publications.

Revelle, W. (2023). *psych : Procedures for psychological, psychometric, and personality research* [Computer software]. https://personality-project.org/r/psych/ https://personality-project.org/r/psych-manual.pdf

Revelle, W., & Condon, D. M. (2018). Reliability. In *The Wiley Handbook of Psychometric Testing* (p. 709-749). https://doi.org/10.1002/9781118489772.ch23

Richmond, L. L., & Zacks, J. M. (2017). Constructing Experience : Event Models from Perception to Action. *Trends in Cognitive Sciences*, *21*(12), 962-980. https://doi.org/10.1016/j.tics.2017.08.005

Sakamoto, Y., Ishiguro, M., & Kitagawa, G. (1984). *Akaike information criterion statistics*. Reidel. https://books.google.be/books?id=w6wQnQEACAAJ

Schwarz, G. (1978). Estimating the Dimension of a Model. *The Annals of Statistics*, *6*(2). https://doi.org/10.1214/aos/1176344136

Streiner, D. L. (2003). Starting at the Beginning : An Introduction to Coefficient Alpha and Internal Consistency. *Journal of Personality Assessment*, *80*(1), 99-103. https://doi.org/10.1207/S15327752JPA8001_18

Taber, K. S. (2018). The Use of Cronbach's Alpha When Developing and Reporting Research Instruments in Science Education. *Research in Science Education*, *48*(6), 1273-1296. https://doi.org/10.1007/s11165-016-9602-2

Thériault, R. (2023). *rempsyc : Convenience functions for psychology* [Computer software]. https://rempsyc.remi-theriault.com

Ushey, K., & Wickham, H. (2023). *renv : Project environments* [Computer software]. https://CRAN.R-project.org/package=renv

Wei, T., & Simko, V. (2021). *R package 'corrplot' : Visualization of a correlation matrix* [Computer software]. https://github.com/taiyun/corrplot

Wickham, H. (2016). *ggplot2 : Elegant graphics for data analysis*. Springer-Verlag New York. https://ggplot2.tidyverse.org

Wickham, H., François, R., Henry, L., Müller, K., & Vaughan, D. (2023). *dplyr : A grammar of data manipulation* [Computer software]. https://CRAN.R-project.org/package=dplyr

Wilke, C. O. (2024). *ggridges : Ridgeline Plots in ggplot2* [Computer software]. https://wilkelab.org/ggridges/

Winter, B., & Wieling, M. (2016). How to analyze linguistic change using mixed models, Growth Curve Analysis and Generalized Additive Modeling. *Journal of Language Evolution*, *1*(1), 7-18. https://doi.org/10.1093/jole/lzv003

Xie, Y. (2015). *Dynamic documents with R and knitr* (2e éd.). Chapman and Hall/CRC. https://yihui.org/knitr/

Xie, Y., Dervieux, C., & Riederer, E. (2020). *R markdown cookbook*. Chapman and Hall/CRC. https://bookdown.org/yihui/rmarkdown-cookbook

Zvonimir, B. (2023). *ggvanced : An R package for creating advanced multivariable plots such as spider/radar charts and parallel plots* [Computer software]. https://github.com/Ringomed/ggvanced
